# Evidence for a functional subdivision of Premotor Ear-Eye Field (Area 8B)

**DOI:** 10.3389/fnbeh.2014.00454

**Published:** 2015-01-30

**Authors:** Marco Lanzilotto, Vincenzo Perciavalle, Cristina Lucchetti

**Affiliations:** ^1^Section of Physiology and Neuroscience, Department of Biomedical Sciences, Metabolic and Neuroscience, University of Modena and Reggio EmiliaModena, Italy; ^2^CSSI, Interdepartmental Facilities Center, University of Modena and Reggio EmiliaModena, Italy; ^3^Section of Physiology, Department of Biomedical Sciences, University of CataniaCatania, Italy

**Keywords:** area 8B, premotor cortex, prefrontal cortex, ear movements, eye movements

## Abstract

The Supplementary Eye Field (SEF) and the Frontal Eye Field (FEF) have been described as participating in gaze shift control. Recent evidence suggests, however, that other areas of the dorsomedial prefrontal cortex also influence gaze shift. Herein, we have investigated electrically evoked ear- and eye movements from the Premotor Ear-Eye Field, or PEEF (area 8B) of macaque monkeys. We stimulated PEEF during spontaneous condition (outside the task performance) and during the execution of a visual fixation task (VFT). In the first case, we functionally identified two regions within the PEEF: a *core* and a *belt*. In the core region, stimulation elicited forward ear movements; regarding the evoked eye movements, in some penetrations, stimulation elicited contraversive fixed-vectors with a mean amplitude of 5.14°; while in other penetrations, we observed prevalently contralateral goal-directed eye movements having end-points that fell within 15° in respect to the primary eye position. On the contrary, in the belt region, stimulation elicited backward ear movements; regarding the eye movements, in some penetrations stimulation elicited prevalently contralateral goal-directed eye movements having end-points that fell within 15° in respect to the primary eye position, while in the lateral edge of the investigated region, stimulation elicited contralateral goal-directed eye movements having end-points that fell beyond 15° in respect to the primary eye position. Stimulation during VFT either did not elicit eye movements or evoked saccades of only a few degrees. Finally, even though no head rotation movements were observed during the stimulation period, we viewed a relationship between the duration of stimulation and the neck forces exerted by the monkey's head. We propose an updated vision of the PEEF composed of two functional regions, core and belt, which may be involved in integrating auditory and visual information important to the programming of gaze orienting movements.

## Introduction

Non-human primates explore the environment, both visually through eye movements and auditorily through eye-ear movements. Sometimes these movements are also accompanied by head movements if the stimuli fall in the periphery or outside the visual field. Together, these movements play an important role in the detection of visual and auditory stimuli from different regions of the surrounding space (Scudder et al., 2002).

The Supplementary Eye Field (SEF), or area F7 (Matelli et al., 1991), and the Frontal Eye Field (FEF), or area 8A (Tehovnik et al., 2000), have been described as participating in gaze shift control, which is the realignment of the line of sight to bring the image of an object of interest to the fovea by means of eye and head movements. Recent evidence suggests, however, that other areas of the dorsomedial prefrontal cortex (DMPFC) and dorsolateral prefrontal cortex (DLPFC) influence the control of gaze shift (Funahashi, 2014). In fact, area 8B, renamed the Premotor Ear-Eye Field (PEEF) (Lucchetti et al., 2008; Bon et al., 2009; Lanzilotto et al., 2013a,b), and the Brodmann Area (BA) 9 are involved in ear- and eye motor control for detecting complex auditory stimuli in the environment (Bon and Lucchetti, 1994, 2006; Lucchetti et al., 2008; Bon et al., 2009; Lanzilotto et al., 2013a,b,c). These data agree with previous studies showing that the DMPFC and DLPFC are involved in auditory-visual discrimination (Fuster et al., 2000) and that their neurons respond to sounds with behavioral salience (Newman and Lindsley, 1976; Bon and Lucchetti, 2006; Lucchetti et al., 2008; Bon et al., 2009; Lanzilotto et al., 2013b). From an anatomical point of view, the auditory and visual properties of these regions could be associated with the connections to the auditory cortex (Romanski et al., 1999a,b; Rauschecker and Romanski, 2011) and with visual areas (Yeterian and Pandya, 2010; Yeterian et al., 2012), which could explain why the FEF and the SEF transform sensory stimuli into gaze shift commands (Chen and Wise, 1995a,b, 1996, 1997; Tu and Keating, 2000; Amador et al., 2004; Chen, 2006; Knight and Fuchs, 2007; Monteon et al., 2010; Knight, 2012). A rabies virus injection into the monkeys' ocular lateral rectus muscles, however, highlighted labeled neurons not only in the FEF and the SEF, but also in areas 8B, 9, and 46 (Moschovakis et al., 2004). This may suggest that gaze shift movements are widely controlled by these regions.

Moreover, there is evidence that several cortical and subcortical regions, which play an important role in saccades generation, are also involved in ear motor control. Supporting this claim, the SEF (Schlag and Schlag-Rey, 1987; Schall et al., 1993; Luppino et al., 2003) and the FEF (Schall et al., 1993) are involved in ear movements, as is the parietal cortex (Cooke et al., 2003; Stepniewska et al., 2005, 2014). Moreover, the intraparietal sulcus (lateral intraparietal area [LIP] and medial intraparietal area [MIP]) has also been demonstrated to play an important role in processing visual and auditory information (Mullette-Gillman et al., 2005). With regard to the subcortical structures, a topographical representation of ear movements was also found in the intermediate and deep lamina of the cat's superior colliculus (Stein and Clamann, 1981). This map of ear movements was found to be in register with the eye movement map, and both maps shared the same axes. The superior colliculus was also characterized by having neurons that integrate auditory and visual information (Stein and Stanford, 2008), and in the inferior colliculus the activity of the hosted auditory neurons was influenced by the eye position (Groh et al., 2001). Considering all this evidence, we could argue that the parietal cortex, the DMPFC and DLPFC, including the SEF and the FEF, and the superior and inferior colliculus have a critical role in the integration of sensory information to generate motor commands such as eye-, head-, and ear-orienting movements.

Finally, as is well-known, all these cortical and subcortical regions are differently influenced by visual attention engagement (Carrasco, 2011). In particular, Bon and Lucchetti (2006) showed that the activity of the auditory neurons in area 8B was modulated by visual attention engagement: they found that about fifty percent of the auditory and auditory-motor neurons which activity was related to the environmental auditory stimuli, were inhibited during the execution of a visual fixation task.

To better understand the role of the DMPFC in gaze shift control, we electrically stimulated area 8B (PEEF) and BA 9 in two macaque monkeys. The stimulation was delivered in two different conditions: the spontaneous condition, i.e., outside the task performance, and during the execution of a visual fixation task (VFT), to verify if the visual attention engagement could affect the evoked movements.

Given the complexity of the findings and the necessity to clearly present them, we described data from BA 9 in a previous paper (Lanzilotto et al., 2013c). Herein, we discuss data from PEEF, showing that this region has specific ear- and eye motor maps and also plays a role in head control. For the first time, considering ear and eye properties, we have identified two functional regions within PEEF that we termed *core* and *belt*.

## Materials and methods

Two adult female macaque monkeys (*Macaca fascicularis*) (3–4 kg, 4–5 years old) were used for these experiments. All phases of the experimental procedure were approved by the local Ethics Committee and followed the standards established by the European Community and Italian law (D.L. 116/92). The project was approved by the Italian National Superior Institute of Health and received authorization from the Italian National Ministry of Health.

### Behavioral methods

The monkeys were preliminarily trained by an apparatus mounted on the monkey's home cage. Each monkey learned to press a bar to illuminate a bi-colored (red/green), light-emitting diode (LED, SIEMENS LS110). The LED, with a diameter of 0.05°, was placed in front of the monkey. After an initial varying red period of 500/5000 ms, the LED turned to green for a fixed period of 500 ms. The task required fixation on the red LED, and the monkey had to release the bar during the green period to receive a liquid reward. After the monkey learned to perform the fixation task in the cage, it was taught to sit in a primate chair inside a Faraday cage and to perform the same task in this new environment. When its performance reached the 80–90% range of correct responses, it was prepared for the eye position measurement and painless head restraint (see Section Surgical Methods). Then, after 1 week, the monkey was trained to complete a VFT with its head restricted. The visual stimuli were presented by homemade software running on a personal computer.

The monkey sat in a primate chair in front of a panel at a distance of 114 centimeters, on which 49 LEDs with a diameter of 0.05° were located. The monkey's head was painlessly restricted by a homemade multipurpose neck robot (MUPRO) (Bon et al., 2002), designed to record both the isometric forces exerted at head level and the head rotations in the horizontal plane. The MUPRO is described in more detail in Section Recording of the Evoked Movements and Data Analysis below.

**Figure 1:**
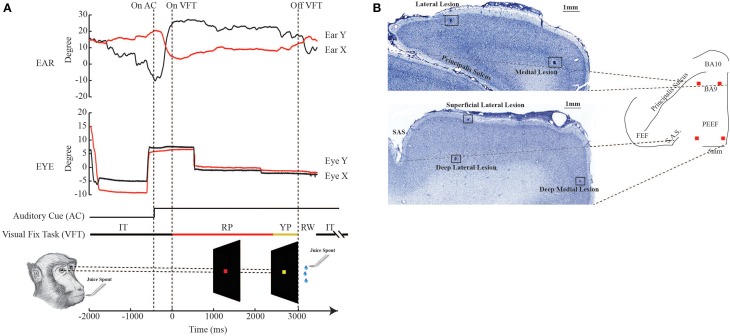
**Experimental design and histological reconstruction. (A)** The bottom figure shows the paradigm of the visual fixation task (VFT). RP: red period; YP: yellow period; RW: reward; IT: intertrial. The upper and middle plots are examples respectively of the normal ear and eye behavior, outside the stimulation period. **(B)** The photomicrographs show the location of the electrolytic lesions in BA 9, rostrally, and on the border between area F7 and PEEF, caudally. Medial lesions are at ~1–2 mm from the inter-hemispheric line, while lateral lesions are at ~8 mm from the inter-hemispheric line.

The monkey performed the VFT in a darkened Faraday cage (which also was a sound-attenuated booth), and a trial began with the ignition of a central red LED (red period). The monkey was required to fixate on this target within an electronic window ranging from 3 to 8 degrees. After a varying red period of 2000–2500 ms, the LED turned yellow for a period of 500 ms (yellow period). After the yellow period, the animal received a few drops of fruit juice or water as a reward. If the monkey's eye moved out of the window, the trial was stopped, and it received neither a reward nor punishment. The red stimulus, representing an instructional pre-cue, required the monkey to maintain the fixation and wait; the yellow stimulus, representing an instructional cue, required it to maintain the fixation and prepare to receive the reward. A 2000 ms inter-trial period followed each trial. An acoustic cue, with an intensity of 40–50 decibels (dB), was switched on at the beginning of each session and switched off at the end, thus signaling to the monkey the beginning and the end of the working period (Figure 1A).

### Surgical methods

Using an aseptic technique and under general anesthesia (Zoletil 10 mg/kg i.m.), a stainless steel cylinder was attached to the animal's skull with three screws, using stereotaxic coordinates, and cemented in place to permit a painless fixation of the head. A scleral search coil was implanted subconjunctivally for the detection of eye movement (Judge et al., 1980).

After the previously described training phase, the monkeys underwent sterile surgery to implant the recording chamber over one hemisphere using stereotaxic coordinates. The inner diameter of the recording chamber was 19 mm, and was vertically oriented to allow a perpendicular approach to the region of interest. During each experimental session, two stainless steel wires were inserted into the neck muscles to monitor the electromyogram (EMG). After each surgical intervention, treatment with antibiotics, cortisone, and analgesics was administered for up to 1 week.

### Physiological methods

After the monkey had achieved 90–95% correct trials in VFT, the experimental sessions began. Each monkey was placed in a primate chair with its head restricted by MUPRO. Quartz-platinum/tungsten microelectrodes were inserted through the dura using a Microelectrode Manipulator System (5-Channel Mini Matrix Thomas Recording). The unit activity was pre-amplified (Preamplifier DPA-4), amplified, and filtered (5-channel Main Amplifier/Filter System MAF-05) to eliminate artifacts from 5 to 75 kHz. The amplified unit activity was monitored using an oscilloscope and was also audio-monitored.

The electrodes were advanced through the entire depth of the cortex. Once the beginning and the end of the cortex were established, we proceeded to stimulate two sites in the same column: one in the deep layers and the other in the superficial layers. We did not stimulate two sites in all penetrations due to the long trains and high current intensities used, since the electrode impedance changed considerably over the course of the experimental session (0.5–1.0 MΩ). Only when the electrodes' properties were almost constant from the beginning to the end of the experimental session did we stimulate two sites in the same penetration. This procedure was used to achieve the best possible empirical experimental approach.

To identify evoked movements at each cortical site studied, stimulation was applied by an S88 stimulator and two PSIU6 stimulus isolation units (Grass Instrument). Long trains of 500, 700, 800, and 1000 ms of duration and 200 μs bipolar pulses were delivered at 300 Hz. Each stimulation pulse was obtained using a biphasic current, where a negative phase was followed by a positive phase to minimize damage that could occur during long-duration stimulation (Graziano et al., 2002a,b). The current was measured by the voltage drop across a 1 kΩ resistor in series with return of the stimulus isolation units. At each cortical site, the stimulating current was injected starting at 20 μA and increased gradually in 10 μA increments until it reached 50 μA, and then gradually increased again until 150 μA, if movements were not evoked below 50 μA. The threshold—in other words, the current at which the movement was evoked 50% of the time at 500 ms of train duration—was determined by two experimenters and then confirmed by offline analysis. If no movements were elicited at 150 μA, the site was defined as non-responsive. The current threshold, described in the Section General Observations, was calculated considering the lowest current value; while the current threshold of the electrode penetrations with two stimulated sites was calculated as the mean of the two sites' thresholds.

Stimulation was applied in the spontaneous condition—that is, outside the task performance—and during the execution of the VFT only in the primary eye position (for details, see Section Behavioral Methods). In this latter condition, the fixed train duration of 500 ms was used during the red period of the VFT (Figure 1 of Lanzilotto et al., 2013c). Stimulation during the VFT was performed to test for attentional effects on the threshold. Five stimulation trials were delivered for each train duration and each current intensity. The total number of stimulation trials for each electrode penetration was variable, since the current threshold could change depending on site. The two experimental conditions were performed in pseudo-random blocks, i.e., sometimes we stimulated first during the VFT and then during the spontaneous condition, while other times we did the opposite. The VFT condition lasted until the monkeys reached 15 correct trials, and in this case the five stimulation trials (e.g., train 500 ms, intensity 50 μA) were delivered pseudo-randomly during the red period. The monkeys received the reward when the trial was correctly performed. After a random pause period, the stimulation occurred in the spontaneous condition. The block of stimulation trials lasted a few minutes, and the five stimulation trials were delivered pseudo-randomly, every 3–4 s. During this phase, no reward was delivered at the end of the stimulation trials. At this point, after a further random pause period, another block of stimulation trials (with different parameters) began. This time, we stimulated first during spontaneous condition and then during the VFT.

Each monkey's behavior was monitored by an infrared video camera placed in front of and above the animal. The experimenters remained outside the Faraday cage, which also was a sound-attenuated booth to provide quieter conditions for the animals.

### Recording of the evoked movements and data analysis

Eye movements were recorded by the search coil technique, using the phase detection method (Remmel, 1984). A coil was chronically implanted subconjunctivally, as previously described in the Section Surgical Methods. The same technique was used for the detection of the contralateral ear movements (Bon and Lucchetti, 1994): a coil was placed on the ear contralateral to the stimulated hemisphere on a daily basis by the same operator to minimize variability in terms of positioning. The ipsilateral ear movements were monitored by means of an infrared video camera. This system allowed us to define the beginning and end of the ear movement. A magnetic field was generated around the monkey's head, and a current proportional to the sin θ (movement amplitude) was induced in both coils. Our search coil system defines a movement in two dimensions (x–y). For eye movements, positive values on the x-axis represent right positions, while negative values represent left positions. Positive values on the y-axis represent upper eye positions, while negative values represent lower eye positions. For ear movements, positive values on the x-axis represent rostral ear positions, while negative values represent caudal ear positions. Positive values on the y-axis represent upper ear positions, while negative values represent lower ear positions.

Finally, forces and/or rotation exerted by the monkey's head in the horizontal plane were detected by MUPRO (Bon et al., 2002), a homemade multipurpose neck robot. MUPRO consists of a mechanical device comprised of a cardan joint, a potentiometer, an electromagnetic brake, and four flexion load cells, which identify the isometric forces applied in four directions of space—forward, backward, right, and left—plus an oleodynamic system that allows head rotation in the horizontal plane between ± 20 degrees. These components are assembled on a column bolted to the primate's chair. An electrical device provides DC power for the potentiometer and the brake. In both animals, the stimulation trials were performed during two different experimental conditions: a spontaneous condition and during the VFT, executed only with the eyes in the primary position. During the spontaneous condition, the starting position for the eye was variable (also true for the ears trial to trial), but during the VFT, the initial eye position was always from the primary position.

Eye and ear movements, LED levels, auditory markers, head forces, rotation signals, and the stimulation marker were sampled at 1 kHz and stored by SuperScope II (GWI) software for data acquisition. Movements were recorded continuously during the experimental session, and kinematic features were analyzed offline using custom MATLAB programs (The MathWorks). MATLAB was also used to perform the statistical analysis.

The analysis sought to define the classes of movements and their topography across the cortical surface. We synchronized the stimulation markers with the x and y components of the ear- and eye-evoked movements and head force signals for the entire duration of the stimulation period.

The evoked eye movements were included for analysis if the peak eye velocity was higher than 30°/s, while the ear movements were included if the peak velocity was higher than 20°/s. The maximal velocity was determined for each evoked movement. Eye onset and offset were then defined as the last points on either side of the peak velocity, before which the tangential velocity fell below 30°/s (Stanford et al., 1996). The onset and offset of the ear movements were calculated using the same method, considering a tangential velocity of 20°/s. This was done because, in general terms, ear movement is slower than eye movement (see Section Results) and this velocity parameter better represented the onset and offset of the ear movements, studied trial by trial. The time range between beginning the stimulation and movement onset was defined as movement latency (in ms). The total time spent during movement was defined as the movement duration (also in ms). Moreover, for each evoked eye- and ear movement, we determined the amplitude of the movement (in degrees), the maximal velocity (in degrees/s), and the mean velocity (in degrees/s). We also included in the analysis only the evoked eye- and ear movements recorded during the stimulation period, which had latencies ranging from 40 to 400 ms to minimize the possibility to include self-initiated movements in the analysis.

**Figure 2:**
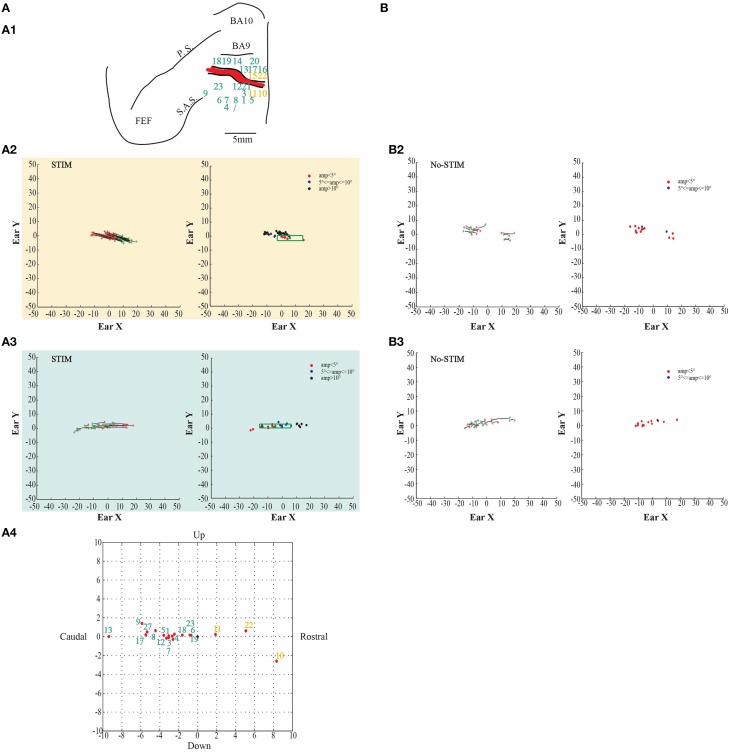
**Example of goal-directed ear movements. (A)** The figures show data from monkey L. **(A1)** Map shows the locations of the electrode penetrations; yellow numbers represent penetrations of the *core* region, while aquamarine numbers represent penetrations of the *belt* region. Red thick line represents a superficial vessel which was avoided during the experimental sessions. FEF: Frontal Eye Field; SAS: Superior Arcuate Sulcus; PS: Principalis Sulcus; BA9: Brodmann Area 9; BA10: Brodmann Area 10. **(A2)** Example of goal-directed forward ear movements in the *core* region; red ^*^: starting position; green ^*^: final position. The plot on the left shows the direction of the ear movements. The plot on the right shows the relationship between starting position and movement amplitude. The green dot in the green square represents the averaged end-point with standard deviation (green square) **(A3)** Example of goal-directed backward ear movements in the *belt* region; **(A4)** Plot shows the averaged end-points, for each penetration, relative to a common starting position. Numbers indicate the electrode penetrations. End-points which have positive values on x-axis indicate forward ear movements. End-points which have negative values on x-axis indicate backward ear movements. **(B)** The figures show self-generated ear movements. **(B2)** The plots show self-generated ear movements sampled outside the stimulation period and from the same experimental session of panel **(A2)**. The plot on the left shows the direction of the ear movements. The plot on the right shows the relationship between starting position and movement amplitude. **(B3)** The plots show self-generated ear movements sampled outside the stimulation period and from the same experimental session of panel **(A3)**. The plot on the left shows the direction of the ear movements. The plot on the right shows the relationship between starting position and movement amplitude.

**Figure 3:**
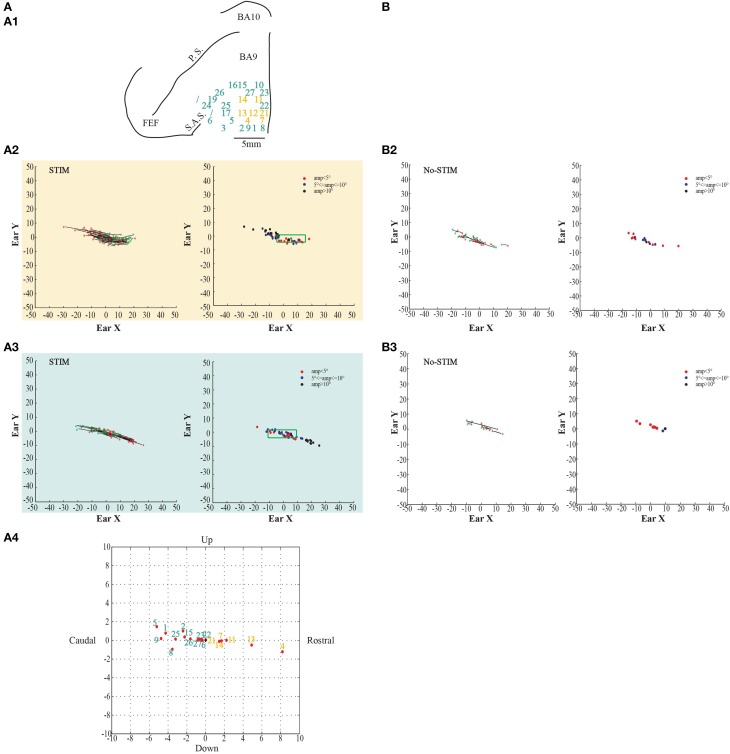
**Example of goal-directed ear movements. (A)** The figures show data from Monkey S. For further information on the figures the reader should refer to the legend of Figure 2.

**Figure 4:**
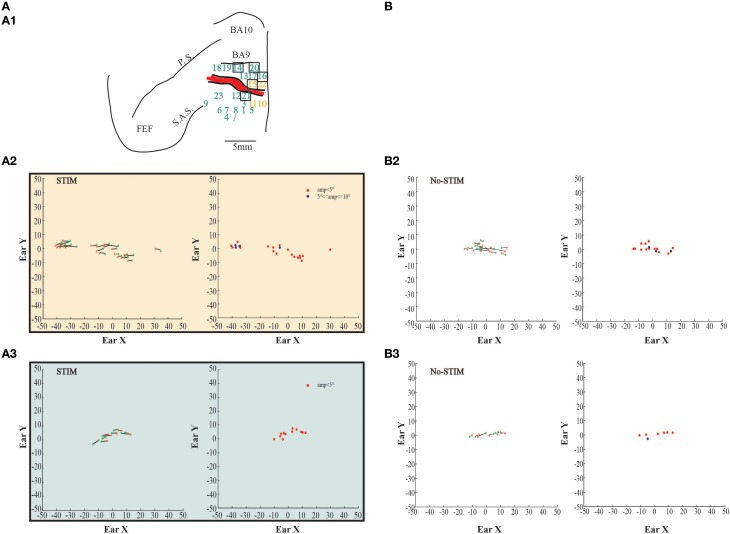
**Example of fixed-vector ear movements. (A)** The figures show data from monkey L. **(A1)** Map shows the locations of the electrode penetrations; numbers in the black squares indicate the penetrations where fixed-vectors were evoked; yellow numbers represent penetrations of the *core* region, while aquamarine numbers represent penetrations of the *belt* region. Red thick line represents a superficial vessel which was avoided during the experimental sessions. FEF: Frontal Eye Field; SAS: Superior Arcuate Sulcus; PS: Principalis Sulcus; BA9: Brodmann Area 9; BA10: Brodmann Area 10. **(A2)** Example of fixed-vector forward ear movements in the *core* region; red ^*^: starting position; green ^*^: ending position. The plot on the left shows the direction of the ear movements. The plot on the right shows the relationship between starting position and movement amplitude. **(A3)** Example of fixed-vector backward ear movements in the *belt* region. **(B)** The figures show self-generated ear movements. **(B2)** The plots show self-generated ear movements sampled outside the stimulation period and from the same experimental session of panel **(A2)**. The plot on the left shows the direction of the ear movements. The plot on the right shows the relationship between starting position and movement amplitude. **(B3)** The plots show self-generated ear movements sampled outside the stimulation period and from the same experimental session of panel **(A3)**. The plot on the left shows the direction of the ear movements. The plot on the right shows the relationship between starting position and movement amplitude.

**Figure 5:**
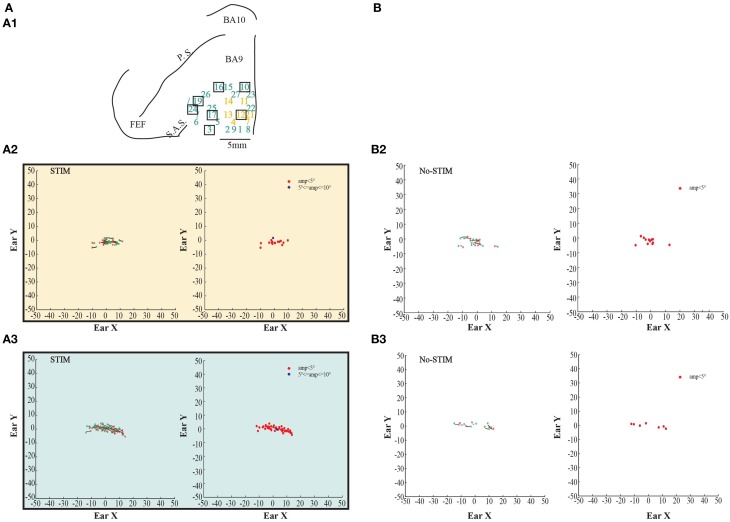
**Example of fixed-vector ear movements. (A)** The figures show data from Monkey S. For further information on the figures the reader should refer to the legend of Figure 3.

**Figure 6:**
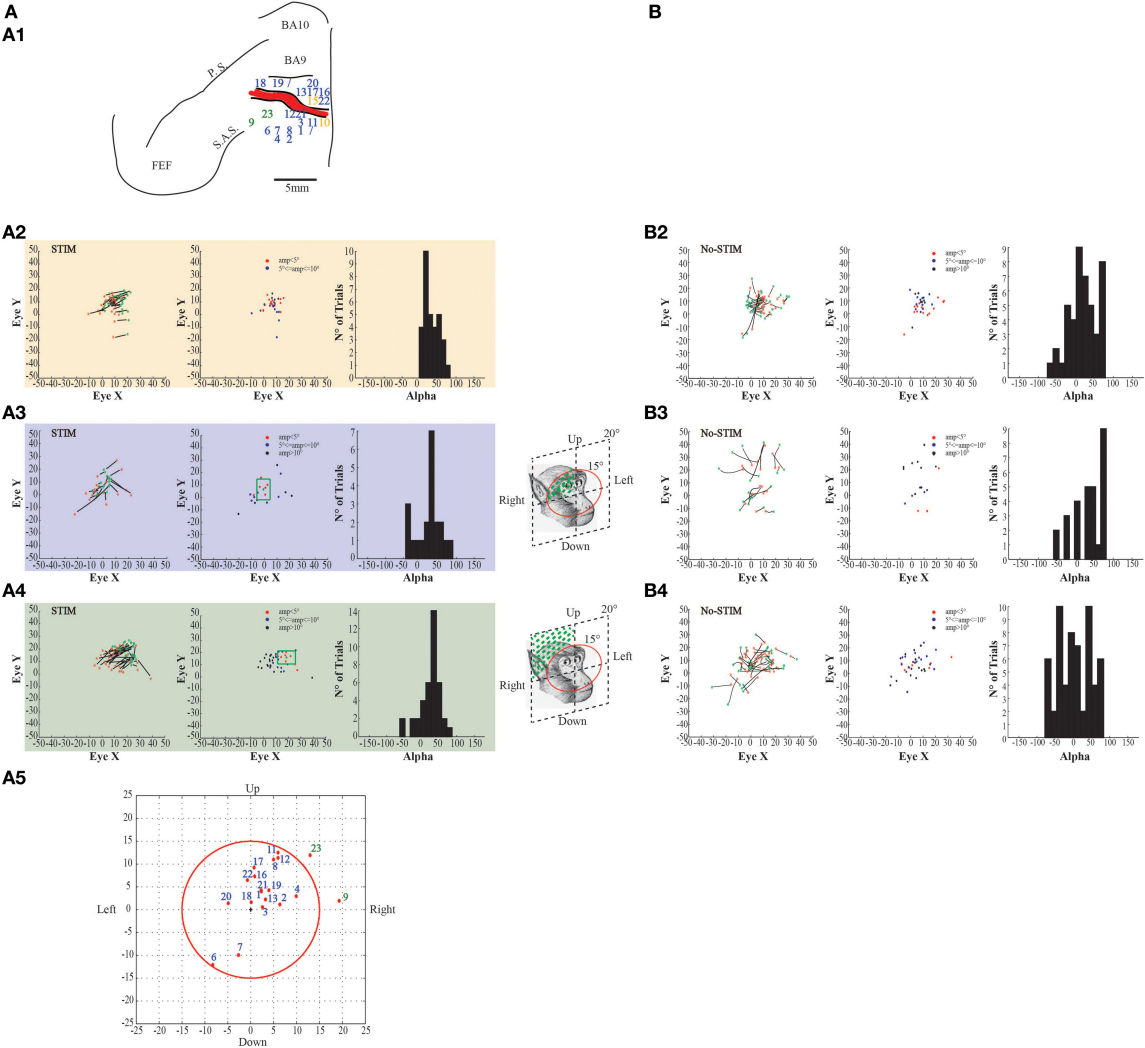
**Example of evoked eye movements. (A)** The figures show data from monkey L. **(A1)** Map shows the locations of the electrode penetrations; yellow numbers represent penetrations where fixed-vector saccades were evoked; blue numbers represent penetrations where contralateral goal-directed eye movements with endpoints falling within 15° in respect to the primary eye position were evoked; greenfinch numbers represent penetrations where contralateral goal-directed eye movements with endpoints falling beyond 15° in respect to the primary eye position were evoked. Red thick line represents a superficial vessel which was avoided during the experimental sessions. FEF: Frontal Eye Field; SAS: Superior Arcuate Sulcus; PS: Principalis Sulcus; BA9: Brodmann Area 9; BA10: Brodmann Area 10. **(A2)** Example of fixed-vector saccades in the *core* region; red ^*^: starting position; green ^*^: ending position. The plot on the left shows the direction of the eye movements. The plot in the middle shows the relationship between starting position and movement amplitude. The plot on the right shows the Alpha values calculated on the basis of the angular coefficient. The values are all positive which indicates that movements go all in the same direction. **(A3)** Example of contralateral goal-directed eye movements with endpoints falling within 15° in respect to the primary eye position in the *belt* region. The plot on the right shows that Alpha values are both positive and negative which indicates that movements have different directions depending by starting position. **(A4)** Example of contralateral goal-directed eye movements with endpoints falling beyond 15° in respect to the primary eye position in the *belt* region. The plot on the right shows that Alpha values are both positive and negative which indicates that movements have different directions depending by starting position. **(A5)** Plot shows the averaged end-points, for each penetration, relative to a common starting position. The red circle indicates the limit of 15°. **(B)** The figures show self-generated eye movements. **(B2)** The plots show self-generated eye movements sampled outside the stimulation period and from the same experimental session of panel **(A2)**. The plot on the left shows the direction of the eye movements. The plot on the middle shows the relationship between starting position and movement amplitude. The plot on the right shows the Alpha values. **(B3)** The plots show self-generated eye movements sampled outside the stimulation period and from the same experimental session of panel **(A3)**. The plot on the left shows the direction of the eye movements. The plot on the middle shows the relationship between starting position and movement amplitude. The plot on the right shows the Alpha values. **(B4)** The plots show self-generated eye movements sampled outside the stimulation period and from the same experimental session of panel **(A4)**. The plot on the left shows the direction of the ear movements. The plot on the middle shows the relationship between starting position and movement amplitude. The plot on the right shows the Alpha values.

**Figure 7:**
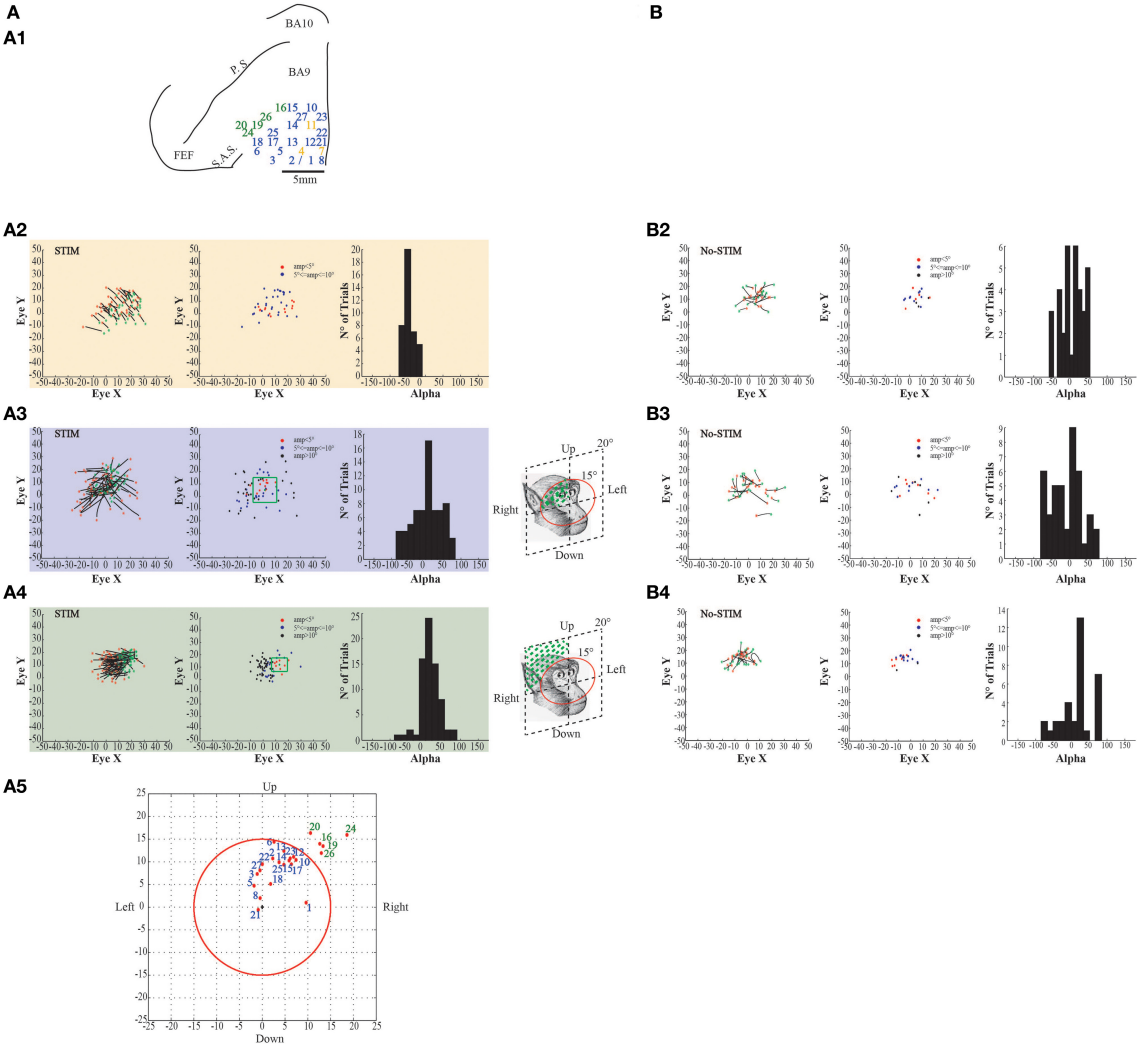
**Example of evoked eye movements. (A)** The figures show data from Monkey S. For further information on the figures the reader should refer to the legend of Figure 6.

**Figure 8:**
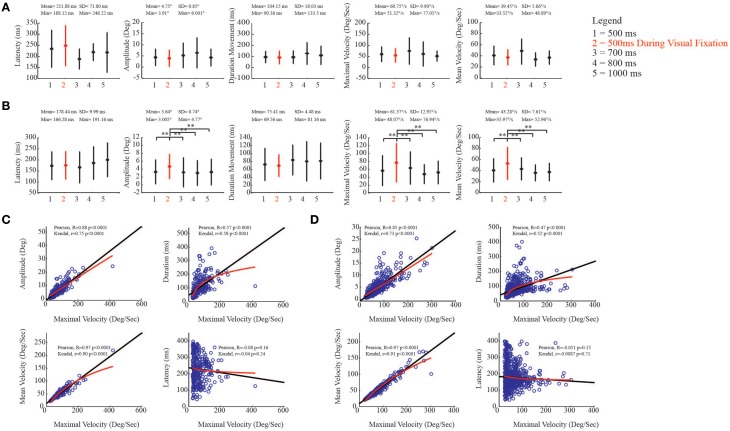
**Kinematic study of the evoked ear movements. (A)** Plots put in relation ear kinematic parameters of the *core* region with the duration of stimulation (numbers on x-axis), during visual fixation task (red), and spontaneous conditions (black). The descriptive statistic shows data which are means ± SEM of 386 determinations; ^**^p < 0.05. Min corresponds to the average minimum value within the 5 conditions. Max corresponds to the average maximum value within the 5 conditions. **(B)** Plots put in relation ear kinematic parameters of the *belt* region with the duration of stimulation (numbers on x-axis), during visual fixation task (red), and spontaneous conditions (black). Latency, movement amplitude, movement duration, maximal and mean velocities are studied. Data are means ± SEM of 974 determinations; ^**^p < 0.05. **(C)** Correlation analysis in the *core* region. Black line represents Pearson correlation, while red line represents non-linear regression (Kendall). Maximal velocity (x-axis) is compared with remaining kinematic parameters (y-axis). **(D)** Correlation analysis in the *belt* region.

**Figure 9:**
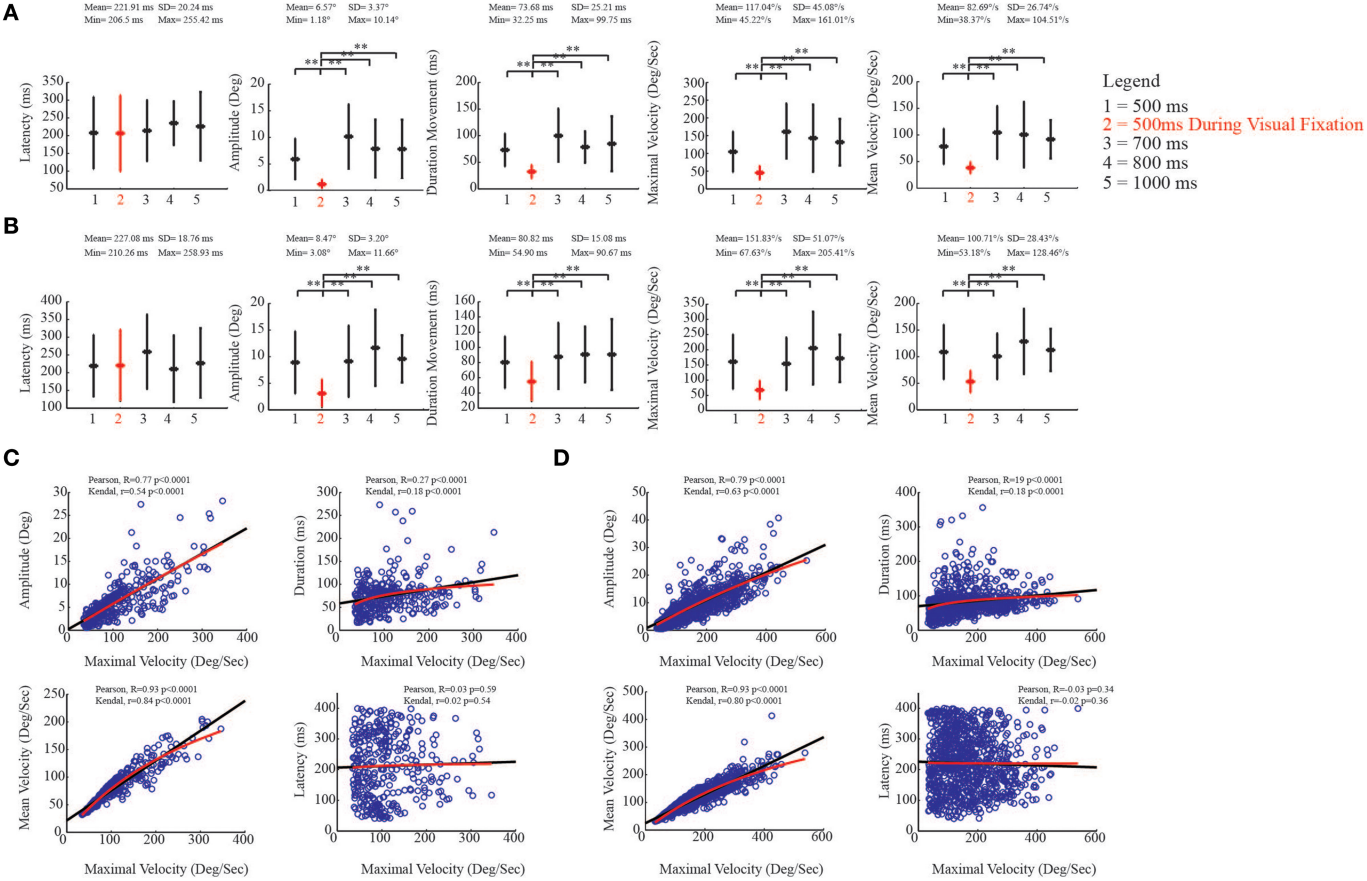
**Kinematic study of the evoked eye movements. (A)** Plots put in relation eye kinematic parameters of the *core* region with the duration of stimulation (numbers on x-axis), during visual fixation task (red), and spontaneous conditions (black). The descriptive statistic shows data which are means ± SEM of 353 determinations; ^**^p < 0.05. Min corresponds to the average minimum value within the 5 conditions. Max corresponds to the average maximum value within the 5 conditions. **(B)** Plots put in relation eye kinematic parameters of the *belt* region with the duration of stimulation (numbers on x-axis), during visual fixation task (red), and spontaneous conditions (black). Latency, movement amplitude, movement duration, maximal and mean velocities are studied. Data are means ± SEM of 993 determinations; ^**^p < 0.05. **(C)** Correlation analysis in the *core* region. Black line represents Pearson correlation, while red line represents non-linear regression (Kendall). Maximal velocity (x-axis) is compared with remaining kinematic parameters (y-axis). **(D)** Correlation analysis in the *belt* region.

We first plotted the x and y components of the evoked movements for each penetration, considering all evoked movements having also different train durations (see Section Results, Figures 2–**5A2,A3** left-most plots; **Figures 6**, **7A2–A4** left-most plots), since the kinematic properties were not affected by the duration of the stimulation (see Section Results, **Figures 8**, **9A,B**). At this point, we tried to establish whether the evoked eye- and ear movements were goal-directed or fixed-vector by referring to the properties described by Bruce et al. (1985) regarding the fixed-vector saccades, and by Schlag and Schlag-Rey (1987) regarding the goal-directed saccades. To accomplish this, we first built plots that showed the relationship between the starting position and the amplitude of the evoked movement. Specifically, we plotted the starting positions of the evoked movements and their averaged end-points for each penetration. The starting positions were painted with one of three different colors: red, if the amplitude of the eye and ear movements was less than 5°; blue, if the amplitude of the eye and ear movements was greater than or equal to 5° and less than or equal to 10°; or black, if the amplitude of the eye and ear movements was greater than 10° (see Section Results, Figures 2–**5A2,A3** middle plots; **Figures 6**, **7A2–A4** middle plots). Whether the starting positions were more concentrated in or around the averaged end-point while the blue and black starting positions were, gradually, more concentrated away from the average end-point, we considered the evoked movements to be goal-directed. On the contrary, if there was not a color gradient because the movement amplitude was almost constant, we classified the evoked movements as fixed-vectors. Secondly, in support of this graphical analysis, we investigated the trajectories of the evoked eye movements, calculating their angular coefficients as follows:

m=(y2−y1)/(x2−x1)

where *m* was the angular coefficient, *y2* was the y-component of the eye final position, *y1* was the y-component of the eye starting position, *x2* was the x-component of the eye final position, and *x1* was the x-component of the eye starting position. At this point, taking into account the angular coefficient, we calculated “Alpha,” which was the angle formed by the straight line with the abscissa axis, as follows:

Alpha=arctan(m)∗180/π

where arctan (m) was the arctangent of the angular coefficient. The Alpha values could be negative or positive, depending on the angular coefficient. If a site of stimulation was characterized by having both positive and negative Alpha values, we classified the movements as goal-directed, since the trajectories were variable. Otherwise, if a site of stimulation was characterized by having Alpha values, either only positive or only negative, we classified the movements as fixed-vectors, since the trajectories were constant (see Section Results, **Figures 6**, **7A2–A4** right-most plots). The same study was also done for the evoked ear movements to establish if the evoked movements were goal-directed or fixed-vectors, even though the analysis of the trajectories was not as informative as the analysis of the eye movements. This is because the eyes have a high motility in all directions (up-down-right-left), while the ears have a high motility principally in only one dimension (rostro-caudal, caudo-rostral).

In order to be sure that the movements—described as “evoked”—were stimulation-evoked (henceforth “stim-evoked”) rather than self-generated, we randomly sampled from the same experimental sessions self-generated eye- and ear movements. In detail, in the recording tracks where stimulation did not occur and the monkeys were free to look around, we sampled twenty 500-ms periods, twenty 700-ms periods, twenty 800-ms periods, and twenty 1000-ms periods. At this point, as was done for the evoked movements, we studied the self-generated eye- and ear movements using the same velocity criterion (eye: onset ≥ 30°/s, offset ≤ 30°/s; ear: onset ≥ 20°/s, offset ≤ 20°/s). We proceeded to investigate the self-generated movements through the same analysis previously described for the evoked movements (see Section Results, Figures 2–**5B2,B3** left-most and right-most plots; **Figures 6**, **7B2–B4** left-most, middle, and right-most plots).

To analyze possible differences in current thresholds within the same monkey, the paired *t*-test was performed. We also performed a Welch's *t*-test (two-sample *t*-test) to observe differences between the monkeys. In addition, in order to assess the effect of stimulation on the evoked ear- and eye movements, during both VFT and spontaneous condition we studied the relationship between the kinematic parameters and the different train durations in both monkeys. One-Way analysis of variance (ANOVA), followed by a *post-hoc* Bonferroni test, were used to multi-compare different experimental conditions. The test was applied for latency, movement amplitude, movement duration, and maximal and mean velocities. To evaluate the relationships between maximal velocity and the other kinematic variables, we first performed the Pearson correlation analysis (R) and then the non-linear correlation analysis (Kendall correlation, r) to better estimate the fit between kinematic variables.

Finally, even though no head rotation movements had been observed during the stimulation period, to establish whether there was a relationship between duration of stimulation and neck activation, we constructed averaged histograms that calculated the maximal and averaged forces exerted by the monkey's head in the horizontal plane during the stimulation period. Because there were no visible head rotation movements, we were not able to calculate latency, head movement duration, and maximal and mean velocities. Data were presented as mean ± standard error of the mean (SEM). To analyze differences and to multi-compare different experimental conditions, One-Way ANOVA followed by a *post-hoc* Bonferroni test were used. Values of *p* < 0.05 were considered statistically significant.

### Histological reconstruction

At the end of the experiments, marking lesions (D.C., 10 μA, 15 s) were made around the stimulated area, medially (~1 mm) and laterally (~8 mm), with respect to the midline (Figure 1B). The animals were then perfused through the left ventricle with 0.9% NaCl physiological saline, followed by 4% formalin. The brains were removed and stored for 3 days in a 10% glycerol and 2% dimethyl sulfoxide solution. The brains were stored for an additional 3 days in a 20% glycerol and 2% dimethyl sulfoxide solution. Later, the brains were frozen in pentane at −80°C, serially sectioned at 60 μm, mounted on slides, and stained with thionin. Slides were examined under light microscopy to identify the marking lesions. Sections presenting the marking lesions were plotted, and the maps were reconstructed.

## Results

### General observations

In this study, we used long train intracortical microstimulation (LT-ICMS), a duration range of 500/1000 ms, and a current intensity up to 150 μA in an attempt to evoke complex movements. Stimulation occurred in two different conditions: in spontaneous condition and during the red period of the VFT. Altogether, 50 electrode penetrations (23 Monkey L; 27 Monkey S) were performed in the PEEF of the two left hemispheres of the macaque monkeys. We stimulated some sites twice, once in the deep layers and the other in the superficial layers, and, as predicted, in some cases we did not evoke movements in the superficial layers. For details regarding the anatomical location of the electrode penetration, the sites' depth from the beginning of the cortex, and the current thresholds, see Tables 1A,B. All the stimulated sites were considered in the analysis. Contralateral ear movements, eye movements, and neck activation were evoked by stimulation in both monkeys during spontaneous condition. Eye movements were difficult to evoke, however, if the monkeys fixated spontaneously on regions of the space during the stimulation period. For this reason, we needed to repeat the stimulation several times while the monkeys gazed in a variety of directions in order to evoke eye movements. When the stimulation occurred during VFT, we still observed evoked ear movements. On the contrary, in this experimental condition, even if we increased the current intensity up to 150 μA, in many cases we were not able to elicit eye movements and only sometimes were able to evoke saccades of a few degrees. Since we used current intensities up to 150 μA and we were not able to reach the 50% mark for the evoked trials, we could not establish the current thresholds in the VFT condition for the eye movements. On the other hand, the current thresholds for the evoked ear movements did not show differences between spontaneous condition and VFT.

**Table 1:** **(A) Table represents the current intensity thresholds for the evoked eye and ear movements in monkey L. Moreover, the depth from the beginning of the cortex is presented for each stimulated site. (B) Table shows the same information for monkey S. Each number represents the electrode penetration number. Italic numbers represent electrode penetrations where twice stimulation was done. The symbol “/” indicates no evoked movements**.

**Electrode penetration**	**Depth from the beginning of the cortex (mm)**	**Current threshold eye (μA)**	**Current threshold ear (μA)**
**(A)**			
1	0.558	50	30
2	0.232	50	/
3	0.400	30	30
4	0.350	30	30
5	0.371	/	30
6	0.676	30	30
7	0.650	30	30
8	1.066	30	30
9	0.815	30	30
10	0.507	20	30
11	0.505	30	30
*12*	1.602	90	90
	0.802	90	/
13	0.502	70	50
*14*	0.487	/	90
	0.187	/	/
15	0.357	70	50
*16*	0.207	/	90
	0.027	100	100
17	0.165	70	70
*18*	0.485	90	70
	0.099	/	70
19	0.500	90	70
20	0.330	50	70
21	0.200	30	30
22	0.593	50	50
23	0.456	50	70
**(B)**			
1	1.000	30	30
2	0.532	50	50
*3*	0.479	30	30
	0.029	70	/
4	0.557	50	20
5	0.351	50	20
6	0.900	70	50
7	0.304	20	20
8	0.753	20	20
9	0.621	/	30
10	0.625	30	40
11	0.570	50	50
12	0.511	50	50
13	0.478	50	20
*14*	0.924	70	70
	0.245	50	70
**(B)**			
*15*	0.471	50	30
	0.069	50	30
*16*	0.395	70	50
	0.035	50	50
*17*	0.377	50	50
	0.100	50	50
*18*	0.556	30	/
	0.056	50	/
19	0.421	50	70
20	0.355	50	/
*21*	0.920	50	/
	0.362	50	70
*22*	(500 ms) 0.404	/	/
	(1000 ms) 0.404	30	30
23	0.426	50	70
24	0.458	30	70
25	0.395	50	20
26	0.150	30	70
27	0.184	50	50

Despite this, considering the direction of the evoked ear movement and eye movement properties in the spontaneous condition, we identified two functional regions within the PEEF, which we termed *core* and *belt*.

### Functional differences between the core and the belt in spontaneous condition

In both monkeys, stimulation of the core region elicited contralateral forward ear movements for a medio-lateral extension of about 2–4 mm with regard to the inter-hemispheric line, and a rostro-caudal extension of about 4 mm (Figures 2, 3A1, yellow penetrations). The evoked ear movements in this region were classified as forward movements because the end-points were located rostrally in respect to the starting position (Figures 2, 3A2, left plots). Moreover, these movements were defined as goal-directed movements because the amplitude of the evoked movements was strictly dependent on the starting position. In fact, if the monkey's ear starting position was caudal at the time of stimulation, we obtained larger forward movements. In contrast, if the monkey's ear starting position was rostral at the time of stimulation, we obtained smaller forward movements (Figures 2, 3A2, right plots). Clear differences were found between stim-evoked and self-generated movements regarding the direction and the amplitude of the movements (Figures 2, 3B2). All around the core region, stimulation elicited contralateral backward ear movements in both monkeys, and we named this second region belt (Figures 2, 3A1, aquamarine penetrations). The evoked ear movements were classified as backward movements because the end-points were located caudally in respect to the starting position (Figures 2, 3A3, left plots). Moreover, these movements were defined as goal-directed movements because the amplitude of the evoked movements was strictly dependent on the starting position. In fact, if the monkey's ear starting position was rostral at the time of stimulation, we obtained larger backward movements. In contrast, if the monkey's ear starting position was caudal at the time of stimulation, we obtained smaller backward movements (Figures 2, 3A3, right plots). Clear differences were found between stim-evoked and self-generated movements regarding the direction and the amplitude of the movements (Figures 2, 3B3). It was possible to show a motor map for the evoked ear movements in both monkeys and to plot the average end-points for each penetration relative to a common starting point (Figures 2, 3A4). Finally, in 12 of 50 penetrations (Figures 4, 5A1, penetrations in the square), we were not able to identify a common end-point for the evoked movements; moreover, the amplitude was almost constant. These evoked ear movements could have properties of fixed-vectors. One penetration for each monkey was located in the core region, where forward ear movements were evoked (Figures 4, 5A2). Clear differences were found between stim-evoked and self-generated movements regarding the direction and the amplitude of the movements (Figures 4, 5B2). The remaining penetrations were located in the belt region, where backward ear movements were evoked (Figures 4, 5A3). Clear differences were found between stim-evoked and self-generated movements regarding the direction and the amplitude of the movements (Figures 4, 5B3).

In the same sites where contralateral ear movements were elicited, eye movements were also evoked. Stimulation in the core region of two penetrations in monkey L and three penetrations in monkey S evoked fixed-vector saccades (Figures 6–7A1, yellow penetrations). They were defined as fixed-vector saccades because, firstly, there was not a common end-point (Figures 6–7A2, left plots); secondly, the amplitude of the evoked eye movements was almost constant (Figures 6–7A2, middle plots); and thirdly, the trajectory (represented by Alpha) was either only positive or only negative in all trials (Figures 6–7A2, right plots), which means that the evoked movements always had the same general direction. In both monkeys, however, the fixed-vectors saccades were contraversive to the stimulated hemisphere. The amplitude of the fixed-vector saccades was, on average, 5.14° ± 3.29°. Clear differences were found between stim-evoked and self-generated movements regarding the direction and the amplitude of the movements (Figures 6–7B2).

Stimulation of the surrounding penetrations (Figures 6–7A1, blue penetrations) elicited principally contralateral goal-directed movements that fell within 15° in respect to the primary eye position (Figures 6–7A3, left-most plots). Stimulation of 10/40 penetrations elicited ipsilateral goal-directed movements (Figures 6–7A5). We considered these goal-directed movements because: firstly, the amplitude of the evoked movements was strictly dependent on the starting position. For example, if the monkey maintained its eyes around or at the end-point, we obtained a smaller or no saccade; but if the monkey's eye starting position was located away from the end-point, we obtained a larger saccade (Figures 6–7A3, middle plots). Secondly, the trajectories (represented by Alpha) had both negative and positive values (Figures 6–7A3, right-most plots), meaning that the evoked movements followed variable directions depending on the starting position. Clear differences were found between stim-evoked and self-generated movements regarding the direction and the amplitude of the movements (Figures 6–7B3). Finally, the stimulation of the lateral edge of the belt region (Figures 6–7A1, greenish penetrations) elicited contralateral goal-directed movements which fell beyond 15° in respect to the primary eye position (Figures 6–7A4, left-most plots). The amplitude of these larger evoked movements was also dependent on the starting position (Figures 6–7A4, middle plots), and the trajectories had both negative and positive values (Figures 6–7A4, right-most plots). Clear differences were found between stim-evoked and self-generated movements regarding the direction and the amplitude of the movements (Figures 6–7B4). Finally, a motor map for the evoked eye movements showing the average end-points for each penetration in both monkeys was observed (Figures 6–7A5).

The Welch's *t*-test did not show any significant difference between monkeys regarding the current thresholds; similarly, the paired *t*-test did not show any significant difference within the same monkey. Specifically, in monkey L, ear movements were evoked using a current threshold of 50 ± 23.09 μA [min = 30 μA; max = 90 μA], while in monkey S, using a current threshold of 43.20 ± 19.09 μA [min = 20 μA; max = 70 μA]. No significant differences were found between monkeys [*t*_(45)_ = 1.10, *p* = 0.27]. Similarly, in monkey L, eye movements were evoked using a current threshold of 51.90 ± 25.02 μA [min = 20 μA; max = 100 μA], while in monkey S, using a current threshold of 43.08 ± 12.25 μA [min = 20 μA; max = 70 μA]. No significant differences were found between monkeys [*t*_(45)_ = 1.58, *p* = 0.12]. Finally, no significant differences were found within the same monkey between ear- and eye thresholds [monkey L, *t*_(20)_ = −0.21, *p* = 0.83; monkey S, *t*_(24)_ = −0.08, *p* = 0.93]. Even though no significant differences were found regarding current thresholds, we observed that the stimulation needed lower current thresholds in the caudal penetrations to evoke ear- and eye movements, while higher current thresholds in the rostral penetrations were needed (Tables 1A,B).

## Kinematic differences between the core and the belt

### Evoked ear movements: spontaneous condition vs. VFT

In order to assess the effect of stimulation on the evoked ear movements, during both VFT and spontaneous condition, we studied the relationship between the kinematic parameters and the different train durations. One-Way ANOVA, followed by a *post-hoc* Bonferroni test, were used to multi-compare different experimental conditions. With regard to the ear, we found that in the core region, the latency of the evoked movements was on average 211.88 ± 71.80 ms (Figure 8A, left-most plot). Moreover, we found that movement amplitude [*F*_(4, 367)_ = 0.41, *p* = 0.80], movement duration [*F*_(4, 367)_ = 0.69, *p* = 0.59], maximal velocity [*F*_(4, 367)_ = 1.81, *p* = 0.13], and mean velocity [*F*_(4, 367)_ = 1.99, *p* = 0.1] showed no significant differences when stimulation was delivered during VFT rather than during spontaneous condition (Figure 8A). Similarly, the latency of the evoked ear movements in the belt region was on average 178.44 ± 9.99 ms (Figure 8B, left-most plot). More interestingly, we found that in the belt region, the amplitude of the evoked movements [*F*_(4, 952)_ = 3.22, *p* = 0.0122], maximal velocity [*F*_(4, 952)_ = 11.44, *p* < 0.0001], and mean velocities [*F*_(4, 952)_ = 13.19, *p* < 0.0001] showed values significantly higher when stimulation was delivered during VFT than during spontaneous condition. For the movement duration, no significant differences were found between VFT and spontaneous condition [*F*_(4, 952)_ = 0.86, *p* = 0.48] (Figure 8B).

To evaluate the relationships between maximal velocity and the other kinematic variables in spontaneous condition, we performed a Pearson correlation and then a non-linear correlation (Kendal) analysis to show the best fit in both core and belt regions. With regard to the core region, maximal velocity and movement amplitude were positively correlated [Pearson, *R* = 0.88 *p* < 0.0001; Kendal, *r* = 0.75 *p* < 0.0001]. The correlation was also positive between maximal velocity and movement duration, even though there was a plateau around 200 ms, and non-linear regression revealed a logarithmic trend as best fit [Pearson, *R* = 0.57 *p* < 0.0001; Kendal, *r* = 0.58 *p* < 0.0001]. The correlation between maximal velocity and mean velocity was also positive [Pearson, *R* = 0.97 *p* < 0.0001; Kendal, *r* = 0.90 *p* < 0.0001], while maximal velocity and latency were not significantly correlated [Pearson, *R* = −0.08 *p* = 0.16; Kendal, *r* = −0.04 *p* = 0.24] (Figure 8C). Similar regression patterns were also found for the belt region (Figure 8D; see figure for statistical values).

### Evoked eye movements: spontaneous condition vs. VFT

As was done for the evoked ear movements, we studied the kinematic parameters of the evoked eye movements and investigated the relationship between maximal velocity and other kinematic variables. For eye movements, we found that the latency in the core region was on average 221.91 ± 20.24 ms (Figure 9A, left-most plot). Moreover, we found that movement amplitude [*F*_(4, 350)_ = 5.33, *p* < 0.0001], movement duration [*F*_(4, 350)_ = 3.56, *p* = 0.0072], maximal velocity [*F*_(4, 350)_ = 5.40, *p* < 0.0001], and mean velocity [*F*_(4, 350)_ = 4.70, *p* = 0.001] showed significantly decreased values when stimulation was delivered during VFT rather than during spontaneous condition (Figure 9A). Similar to the core region, the latency of the evoked eye movements in the belt region was, on average, 227.08 ± 18.76 ms (Figure 9B, left-most plot). We also found that, in the belt region, movement amplitude [*F*_(4, 982)_ = 8.60, *p* < 0.0001], movement duration [*F*_(4, 982)_ = 6.18, *p* < 0.0001], maximal velocity [*F*_(4, 982)_ = 9.13, *p* < 0.0001], and mean velocity [*F*_(4, 982)_ = 8.51, *p* < 0.0001] showed values that significantly decreased when stimulation was delivered during VFT rather than during spontaneous condition (Figure 9B).

To evaluate the relationships between maximal velocity and the other kinematic variables in spontaneous condition, we performed the Pearson correlation and then the non-linear correlation (Kendal) analysis to show the best fit in both core and belt regions. With regard to the core region, as expected, maximal velocity and movement amplitude were positively correlated [Pearson, *R* = 0.77 *p* < 0.0001; Kendal, *r* = 0.54 *p* < 0.0001]. The correlation between maximal velocity and movement duration was also positive, and non-linear regression revealed a logarithmic trend as best fit [Pearson, *R* = 0.27 *p* < 0.0001; Kendal, *r* = 0.18 *p* < 0.0001]. The correlation between maximal velocity and mean velocity was positive [Pearson, *R* = 0.93 *p* < 0.0001; Kendal, *r* = 0.84 *p* < 0.0001], while maximal velocity and latency were not significantly correlated [Pearson, *R* = 0.03 *p* = 0.59; Kendal, *r* = 0.02 *p* = 0.54] (Figure 9C). A similar regression pattern was also found for the belt region (Figure 9D; see figure for statistical values).

## Relationship between duration of stimulation and neck activation

**Figure 10:**
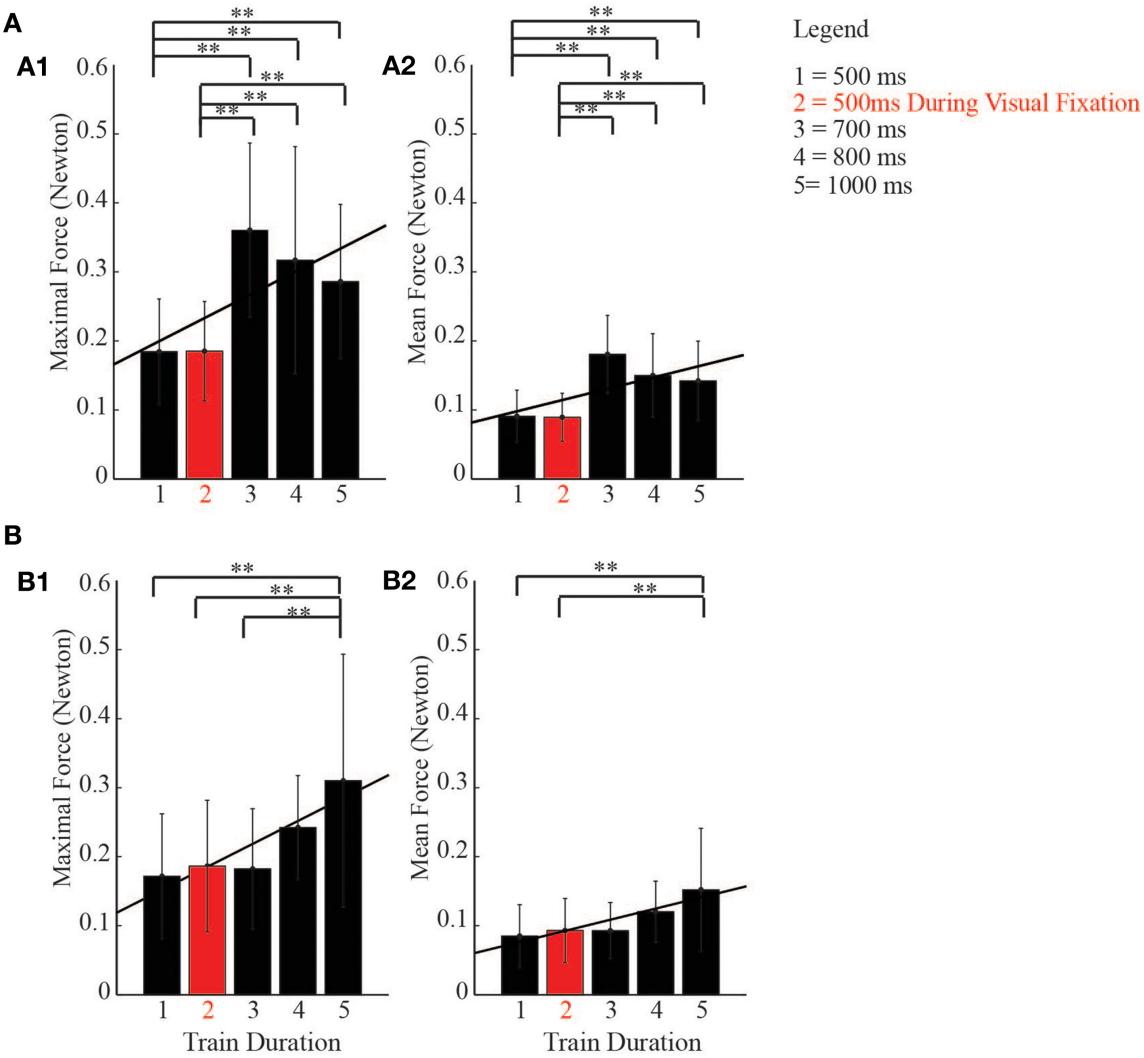
**Relationship between duration of stimulation and neck activity. (A)** histograms show a linear correlation (Pearson, black line) between duration of stimulation and neck forces exerted by the monkeys' head during the stimulation of the *core* region. **(B)** histograms show a linear correlation (Pearson, black line) between duration of stimulation and neck forces exerted by the monkeys' head during the stimulation of the *belt* region, ^**^ indicates *p* < 0.05.

Even though no head rotation movements coordinated with ear- and eye movements were observed during the stimulation period, to establish whether there was a relationship between duration of stimulation and the development of neck forces recorded by MUPRO, we constructed averaged histograms; on the x-axis we plotted the train durations, while on the y-axis we plotted the maximal and averaged forces exerted by the monkey's head during the stimulation period. One-Way ANOVA, followed by a *post-hoc* Bonferroni test, were used to multi-compare different experimental conditions. Moreover, a Pearson correlation showed the linear relationship between the duration of stimulation and neck forces exerted by the monkey's head. The test performed on the maximal forces recorded during the stimulation of the core region showed significant differences between a basic condition of 500 ms and longer train durations [*F*_(4, 75)_ = 6.89, *p* < 0.0001]. The Pearson correlation showed a linear correlation (*r* = 0.67, *p* = 0.022) (Figure 10A1). A similar result was also observed for the averaged forces [*F*_(4, 75)_ = 7.20, *p* < 0.0001; Pearson correlation: *r* = 0.65, *p* = 0.023] (Figure 10A2). The same phenomenon was also seen in the belt region. In fact, the test showed significant differences between the basic condition of 500 ms and longer train durations [*F*_(4, 248)_ = 12.97, *p* < 0.0001]. In support, the Pearson correlation showed a linear correlation (*r* = 0.91, *p* = 0.0034) (Figure 10B1). A similar result was also observed for the averaged forces [*F*_(4, 248)_ = 12.41, *p* < 0.0001; Pearson correlation: *r* = 0.92, *p* = 0.0026] (Figure 10B2).

## General discussion

There has been an ongoing debate regarding the presence of an eye movement representation rostral to the SEF. Some researchers have evoked eye movements (Mitz and Godschalk, 1989; Bon and Lucchetti, 1994), while others (Matsuzaka et al., 2012) have described that stimulation of the caudal prefrontal cortex, which should correspond to area 8B (PEEF), did not elicit eye movements. This non-excitability was used to distinguish the prefrontal cortex from the pre-SMA and the SEF rostro-caudally. First, it is likely that different stimulation parameters could give different results. Second, in the paper by Matsuzaka et al. (2012) it is not clear from the method's description if the stimulation was delivered in spontaneous condition or during the task performance. If the stimulation occurred during the task it is likely that eye movements were not elicited. In fact, in the present article we found that stimulation during the VFT either did not evoke eye movements or evoked saccades of a few degrees. We also found that in spontaneous condition, stimulation did not elicit eye movements if the monkeys fixated spontaneously on regions of the space during the stimulation period. For this reason, we needed to repeat the stimulation several times while the monkey gazed in a variety of directions in order to evoke eye movements. Similar results were found by Mitz and Godschalk (1989) when stimulating a region corresponding to the PEEF. Moreover, this phenomenon is in accord with Chapman and Corneil (2014), who found that short ICMS of SEF did not evoke saccades during the task performance, even if it influenced the percentage of errors and the reaction times of the anti-saccades and pro-saccades.

Because the monkeys fixated spontaneously on regions of the space—which then meant that we needed to repeat the stimulation several times while the monkeys gazed in a variety of directions—it is reasonable to expect to find higher latencies in PEEF in comparison to other similar studies. For example, stimulation of the SEF elicited eye movements having latencies ranging from 40 to 160 ms (Chapman et al., 2012) and from 60 to 120 ms (Chapman and Corneil, 2014). Yang et al. (2008) found when stimulating the dorsomedial frontal cortex (DMFC) that the latency of the evoked movements was about 80–86 ms, accompanied by a small standard deviation, even though they found also that the latency change was not correlated with the change in endpoint and saccade metrics or dynamics. Moreover, Bon and Lucchetti (1994) found, after short ICMS of area 8B, that the latency of both ear- and eye-evoked movements ranged from 40 to 200 ms. Unfortunately, although Mitz and Godschalk (1989) stimulated a region corresponding to the PEEF, they did not describe eye movement latencies in their paper.

The fact that adjacent regions show different latency values supports the hypothesis for the presence of a different field located rostrally to the SEF, recently called Premotor Ear-Eye Field (PEEF) (Bon et al., 2009; Lanzilotto et al., 2013a,b). Our recent results, in fact, are in accord with previous results (Bon and Lucchetti, 1994) that described eye and ear movements in area 8B. With regard to eye movements, Bon and Lucchetti (1994) showed that short ICMS of area 8B principally elicited fixed-vector saccades, in conflict with our present findings, where we show both fixed-vector and goal-directed saccades, with a prevalence of goal-directed saccades. This discrepancy could be, firstly, because Bon and Lucchetti (1994) stimulated a smaller region than we explored herein; and secondly, because they used shorter durations of stimulation. In fact, previous research showed that a short duration of stimulation truncates movements prematurely (Graziano et al., 2002b). When the stimulation duration is extended to permit movement completion, complex movement sequences, rather than muscle twitches, can be observed. In support, Tehovnik and Sommer (1997) found that to evoke saccades readily from the dorsomedial frontal cortex (DMFC), train durations greater than 200 ms were needed, while for the FEF, durations of less than 100 ms were sufficient.

Besides evoking fixed- and goal-directed eye movements, we herein showed that stimulation of PEEF also elicited ear movements, which were topographically represented within PEEF. Considering the direction of the evoked ear movements and the saccades properties, we identified two functional regions within the PEEF, which we termed *core* and *belt*.

Stimulation of the core region elicited forward ear movements; in some penetrations, contraversive fixed-vector eye movements with a mean amplitude of 5.14°; while in other penetrations, principally contralateral goal-directed eye movements having end-points that fell within 15° in respect to the primary eye position. The fixed-vector saccades that we found in the core region show properties similar to the saccades described by Bruce et al. (1985) in the ventrolateral portion of FEF, and similar to the saccades described by Bon and Lucchetti (1994) in area 8B. Conversely, stimulation of the belt region elicited backward ear movement; in some penetrations, principally contralateral goal-directed eye movements having end-points that fell within 15° in respect to the primary eye position. In the lateral edge of the investigated region, contralateral goal-directed eye movements had end-points that fell beyond 15° in respect to the primary eye position. One might think that the evoked ear- and eye movements could be self-generated rather than stim-evoked movements. One might also think that the goal-directed eye movements, having end-points that fell within 15° in respect to the primary eye position, could be an effect of a re-centering bias due to the presence of the juice spout in front of the monkeys. To help alleviate concerns, we studied only movements with latencies ranging from 40 to 400 ms to minimize the possibility of including self-initiated movements. Moreover, we studied the self-generated eye- and ear movements, generated by the monkeys themselves outside the stimulation period. First, we observed that the self-generated movements had random directions and amplitudes, as opposed to the eye and ear movements that we considered stim-evoked. Second, we excluded the possibility of a re-centering bias because we never rewarded the monkeys after the stimulation in spontaneous condition, and because different end-points were found in different stimulated regions. Nevertheless, we cannot exclude that self-generated movements might have been unintentionally included in our analysis.

Other brain regions have been shown to have a role in gaze shift, with particular reference to the FEF (Bizzi and Schiller, 1970; Azuma and Suzuki, 1984; Bruce and Goldberg, 1985; Bruce et al., 1985; Azuma et al., 1988; Blanke et al., 1999; Chen, 2006; Elsley et al., 2007; Knight and Fuchs, 2007; Monteon et al., 2010, 2013; Knight, 2012; Funahashi, 2014), SEF (Bon and Lucchetti, 1992; Chen and Wise, 1995a,b, 1997; Amador et al., 2004; Chen and Walton, 2005; Chapman et al., 2012; Chapman and Corneil, 2014) and superior colliculus (Stryker and Schiller, 1975; Stein and Clamann, 1981; Stanford et al., 1996; Freedman and Sparks, 1997; Corneil et al., 2002; Populin et al., 2004). In support, superior colliculus hosts neurons integrating visual and auditory information (Stein and Stanford, 2008) and its stimulation in cats revealed the presence of an ear motor map overlapping with an eye motor map (Stein and Clamann, 1981). Similar results are found in the present paper, which highlights that the PEEF plays an important role in the ear- and eye motor control. Moreover, the patterns of the eye- and ear movements observed in PEEF could be similar to those observed recently in BA 9 (Lanzilotto et al., 2013c). There are many difference between these two frontal cortical areas, however. First of all, in PEEF we identified two functional regions, the core and the belt, where different eye and ear motor maps were described, while in BA 9 we identified only backward ear movements and goal-directed eye movements directed principally toward the central part of the visual field. Secondly, the thresholds of current to evoke ear and eye movements were different between PEEF and BA 9. In fact, in the PEEF, ear- and eye movements were evoked with an average of ~40 and ~50 μA, respectively, while in the BA 9 ear- and eye movements were evoked with an average of ~50 and ~65 μA, respectively. These different functional aspects, which characterized the rostral BA9 and the caudal PEEF, could be in line with the hypothesis of a functional rostro-caudal gradient of the entire frontal cortex that might reflect a hierarchical organization of the action control (O'reilly et al., 2002; Buckner, 2003; Koechlin et al., 2003; Miller and D'esposito, 2005). In this hypothesis, the more rostral prefrontal areas seem to be involved in the high-level control of the action abstraction. In detail, the rostral areas could exert a top-down control onto caudal lateral frontal areas that, on the other hand, would process lower-level aspects of the action. Following this hypothesis, because we observed the longer latencies of the evoked movements of PEEF and BA 9 compared to the SEF and FEF, and the higher current intensities necessary to evoke movements compared to the SEF and FEF, we could speculate that BA 9 could exert a top-down control onto the PEEF, and both may exert a top-down control onto the SEF and the FEF.

Finally, if we take into account the findings regarding the kinematic analysis of the evoked ear- and eye movements, one could speculate that the neuronal populations of both the core and belt regions could be affected by the visual attention engagement. In fact, in the belt region, the amplitude and the velocity of the ear movements significantly increased when stimulation was delivered during VFT rather than during spontaneous condition. On the contrary, in both the core and belt regions, the amplitude, the duration, and the velocity of the eye movements significantly decreased when stimulation was delivered during VFT rather than during spontaneous condition. These findings could be in line with other studies in which, when a monkey is required to actively fixate on a spot to receive a juice reward, the current threshold to evoke saccades increased 3-fold in the FEF, 16-fold in the dorsomedial frontal cortex, and over 40-fold in V1 (Tehovnik et al., 2003), suggesting that the behavioral state of an animal can override the effects of stimulation on cerebral cortex. But, if the visual attention engagement had affected the neuronal population, we should have observed differences in latencies between different experimental conditions. It did not occur, likely because the variation of the amplitude and the velocity of the evoked movements during the VFT could be due to the effector position. In fact, we found that the evoked ear movements were increased during VFT rather than in spontaneous condition only in the belt region where the stimulation elicited backward ear movements, but not in the core region where stimulation elicited forward ear movements. This could be due to the fact that both monkeys during the VFT moved the ear forward and fixated on the visual target (Figure 1A). Following this logic, the stimulation of the belt increased ear kinematic parameters because the ear starting position was rostral at the time of the stimulation, while the stimulation of the core region did not increase ear kinematic parameters because the ear starting position was already rostral at the time of the stimulation.

### Role for PEEF in head motor control

In both core and belt regions we observed that there was a positive correlation between the duration of the stimulation and the neck activation. It is in accord with a recent interesting finding (Gu and Corneil, 2014) which showed that the transcranial magnetic stimulation of a prefrontal region corresponding to the belt region of the PEEF evokes a polysynaptic neck muscle response that reflects oculomotor activity. This is in accord with our recent hypothesis of a spatial organization of the auditory and visual systems in the DLPFC (Lanzilotto et al., 2013a). Similar and equally interesting results have been recently highlighted from the stimulation of the BA 9 (Lanzilotto et al., 2013c) where the authors also showed a positive correlation between the duration of stimulation and the neck forces exerted by the monkey's head. Surprisingly, in the present paper we never evoked visible head movements coordinated with ear and eye movements. Several results showed that the FEF, the SEF, the parietal cortex, and superior colliculus are involved in eye–head orienting movements (Chen and Tehovnik, 2007). In some cases, head contribution was visible using current intensities exceeding 150 μA (Thier and Andersen, 1998; Tu and Keating, 2000) and in unrestricted head condition (Chen and Walton, 2005), which could be the reason why we did not evoke coordinated ear/eye/head movements. In fact, although we partially released the head during the experimental phase, the MUPRO inertia could resist the evoked head movement. Moreover, we tested the penetration sites with current intensity up to 150 μA to elicit ear- and eye movements. One final reason we did not evoke head orienting movements was likely because of the presence of the juice tube in front of the animal, which could create a bias for the animal to stay to the center. Despite this, we were able to indirectly show the neck activation by analyzing the forces applied by the monkey's head in the horizontal plane during the stimulation period. We observed that in both the core and belt regions, there was a significant development of neck forces when the duration of stimulation became longer, even though no head-orienting movement was visible (Figures 10A,B). Interestingly, at the same train durations, the eye movement reached the highest mean amplitude, about 10–15° (Figures 9A,B). In accordance, as is well-known, when the eyes are centered in the orbits, gaze shifts of <20° are usually completed without any head contribution (Gandhi and Sparks, 2001), while gaze shifts of >20° usually involve a significant contribution of the head (Freedman and Sparks, 1997). Finally, other evidence (Stryker and Schiller, 1975; Chen and Walton, 2005) revealed that increasing the train duration made it easier to evoke head-orienting movements.

### Hypothesis for gaze-shift prefrontal circuits

At this point, altogether the BA 9, the PEEF, the SEF, and FEF could be considered part of a salient network deputed to detect visual and auditory stimuli from different regions of space by means of ear, eye, and head movements. Orienting the eyes toward the central part of the visual field and the ears forward could be a behavior that helps monkeys to detect visual and auditory stimuli from the frontal region of their environment. Conversely, orienting the eyes toward the peripheral part of the visual field and the ears backward could be a behavior that helps monkeys to detect visual and auditory stimuli from the peripheral region. We therefore speculate that the core region and the belt region of the PEEF could be part of parallel circuits with a topographical representation of the surrounding space. A hypothesis of neural circuits devoted to detecting stimuli from central and peripheral regions of the space has been proposed by Schall et al. (1995), which showed that the FEF is topographically innervated by the visual system. The authors demonstrated that the ventral part of the FEF receives visual information from areas where fovea is clearly represented, while the dorsal part of the FEF is connected with areas where the peripheral visual field is represented. Moreover, the SEF also appears to have a topographic organization of its connectivity with the FEF (Schall et al., 1993).

Interestingly, the hypothesis that the PEEF could even play a role in detecting auditory and visual stimuli from different regions of the space is supported by recent anatomical studies (Gerbella et al., 2010; Borra et al., 2013). Two subsectors of ventral prefrontal area 45 (45A and 45B)—for which a role in communication behavior and homology with the human Broca's area has been proposed (Gerbella et al., 2010; Kelly et al., 2010; Hage and Nieder, 2013)—are differentially connected with DMPFC, DLPFC, brainstem preoculomotor structures, basal ganglia, and cerebellar oculomotor loops. Area 45B (Gerbella et al., 2010) is connected with the ventral part of the FEF, where small saccades are represented; with the SEF; with a medial portion of area 8B, which should correspond to the core region identified in the present research; and BA 9. Area 45A (Gerbella et al., 2010) is connected with the rostral and caudal auditory parabelt; with the dorsal part of the FEF, where the largest saccades are represented; with the SEF; with a lateral portion of area 8B, which should correspond to the belt region identified in the present research; and BA 9, where goal-directed saccades are represented (Lanzilotto et al., 2013c). In support, Frey et al. (2014) also found that injection of area 45B shows connections with a small region of the DMFC, which should correspond to the core region of the PEEF, which we identified by stimulation. Finally, Takahara et al. (2012) found connections between area 45 and the DLPFC, including 46d/9/8B/8Ad, F7, and pre-SMA.

Finally, one could also speculate a further and interesting role of these prefrontal circuits in the processing of multimodal communicative signals. Recent results in humans indicate that the hemodynamic activity increases more prominently in the DMPFC when a subject views another subject with a direct gaze rather than with an averted gaze associated to a vocal sound. In contrast, the hemodynamic activity increases in the DLPFC regardless of the subject's gaze direction (Urakawa et al., 2014). Further findings confirm these data (Kampe et al., 2003; Schilbach et al., 2006) and has been also demonstrated that the DMPFC is activated when a subject hears a voice calling the subjects' name (Kampe et al., 2003). For these reasons, a specific role in the perception of face-to-face communication has been assigned to the DMPFC, especially regarding the mutual gaze essential for the social interaction.

Altogether, anatomical and functional evidence supports the hypothesis that different circuits are involved to detect auditory and/or visual stimuli from different regions of the space.

## Conclusion

If we consider the present findings and data from BA 9 (Lanzilotto et al., 2013c), we can speculate that the core and belt regions of the PEEF, BA 9, the SEF, and the FEF could be part of prefrontal circuits with a topographical organization of the surrounding space. The present findings, however, are not in contrast with the known role of the DMPFC and DLPFC in higher cognitive functions such as working memory, planning, and reasoning (Fuster, 1997, 2008). In fact, eye/head movements have an extremely important role for information-seeking in primates, and therefore in the high-level selection of the visual objects (Gottlieb, 2013). Our findings provide a useful basis for guiding future research on the organization of the DMPFC.

### Conflict of interest statement

The authors declare that the research was conducted in the absence of any commercial or financial relationships that could be construed as a potential conflict of interest.

## References

[B1] Amador N. Schlag-Rey M. Schlag J. (2004). Primate antisaccade. II. Supplementary eye field neuronal activity predicts correct performance. J. Neurophysiol. 91, 1672–1689. 10.1152/jn.00138.2003 14645374

[B2] Azuma M. Nakayama H. Sasaki Y. Suzuki H. (1988). Relation between visual input and motor outflow for eye movements in monkey frontal eye field. Behav. Brain Res. 27, 93–98. 10.1016/0166-4328(88)90034-4 3358856

[B3] Azuma M. Suzuki H. (1984). Properties and distribution of auditory neurons in the dorsolateral prefrontal cortex of the alert monkey. Brain Res. 298, 343–346. 10.1016/0006-8993(84)91434-3 6722560

[B4] Bizzi E. Schiller P. (1970). Single unit activity in the frontal eye fields of unanesthetized monkeys during eye and head movement. Exp. Brain Res. 10, 151–158. 10.1007/BF00234728 4985047

[B5] Blanke O. Morand S. Thut G. Michel C. Spinelli L. Landis T.. (1999). Visual activity in the human frontal eye field. Neuroreport 10, 925–930. 10.1097/00001756-199904060-00006 10321461

[B6] Bon L. Lanzilotto M. Lucchetti C. (2009). PEEF: Premotor Ear-Eye Field. A new vista of area 8B, in Prefrontal Cortex: Roles, Interventions and Traumas, eds LoGrasso L. Morretti G. (Portland, OR: Nova Science Publisher), 157–175.

[B7] Bon L. Lucchetti C. (1992). The dorsomedial frontal cortex of the macaca monkey: fixation and saccade-related activity. Exp. Brain Res. 89, 571–580. 10.1007/BF00229882 1644122

[B8] Bon L. Lucchetti C. (1994). Ear and eye representation in the frontal cortex, area 8b, of the macaque monkey: an electrophysiological study. Exp. Brain Res. 102, 259–271. 10.1007/BF00227513 7705504

[B9] Bon L. Lucchetti C. (2006). Auditory environmental cells and visual fixation effect in area 8B of macaque monkey. Exp. Brain Res. 168, 441–449. 10.1007/s00221-005-0197-5 16317576

[B10] Bon L. Lucchetti C. Portolan F. Pagan M. (2002). Equipment note MUPRO: a multipurpose robot. Int. J. Neurosci. 112, 855–868. 10.1080/00207450290025888 12424826

[B11] Borra E. Gerbella M. Rozzi S. Luppino G. (2013). Projections from caudal ventrolateral prefrontal areas to brainstem preoculomotor structures and to basal ganglia and cerebellar oculomotor loops in the macaque. Cereb. Cortex 24, 24. 10.1093/cercor/bht265 24068552

[B12] Bruce C. Goldberg M. (1985). Primate frontal eye fields. I. Single neurons discharging before saccades. J. Neurophysiol. 53, 603–635. 3981231 10.1152/jn.1985.53.3.603

[B13] Bruce C. Goldberg M. Bushnell M. Stanton G. (1985). Primate frontal eye fields. II. Physiological and anatomical correlates of electrically evoked eye movements. J. Neurophysiol. 54, 714–734. 4045546 10.1152/jn.1985.54.3.714

[B14] Buckner R. L. (2003). Functional–anatomic correlates of control processes in memory. J. Neurosci. 23, 3999–4004. 12764084 10.1523/JNEUROSCI.23-10-03999.2003 PMC6741083

[B15] Carrasco M. (2011). Visual attention: the past 25 years. Vision Res. 51, 1484–1525. 10.1016/j.visres.2011.04.012 21549742 PMC3390154

[B16] Chapman B. B. Corneil B. D. (2014). Short-duration stimulation of the supplementary eye fields perturbs anti-saccade performance while potentiating contralateral head orienting. Eur. J. Neurosci. 39, 295–307. 10.1111/ejn.12403 24417515

[B18] Chapman B. Pace M. Cushing S. Corneil B. (2012). Recruitment of a contralateral head turning synergy by stimulation of monkey supplementary eye fields. J. Neurophysiol. 107, 1694–1710. 10.1152/jn.00487.2011 22170964

[B19] Chen L. (2006). Head movements evoked by electrical stimulation in the frontal eye field of the monkey: evidence for independent eye and head control. J. Neurophysiol. 95, 3528–3542. 10.1152/jn.01320.2005 16554500

[B20] Chen L. Tehovnik E. (2007). Cortical control of eye and head movements: integration of movements and percepts. Eur. J. Neurosci. 25, 1253–1264. 10.1111/j.1460-9568.2007.05392.x 17425554

[B21] Chen L. Walton M. (2005). Head movement evoked by electrical stimulation in the supplementary eye field of the rhesus monkey. J. Neurophysiol. 94, 4502–4519. 10.1152/jn.00510.2005 16148273

[B22] Chen L. Wise S. (1995a). Neuronal activity in the supplementary eye field during acquisition of conditional oculomotor associations. J. Neurophysiol. 73, 1101–1121. 7608758 10.1152/jn.1995.73.3.1101

[B23] Chen L. Wise S. (1995b). Supplementary eye field contrasted with the frontal eye field during acquisition of conditional oculomotor associations. J. Neurophysiol. 73, 1122–1134. 7608759 10.1152/jn.1995.73.3.1122

[B24] Chen L. Wise S. (1996). Evolution of directional preferences in the supplementary eye field during acquisition of conditional oculomotor associations. J. Neurosci. 16, 3067–3081. 8622136 10.1523/JNEUROSCI.16-09-03067.1996 PMC6579060

[B25] Chen L. Wise S. (1997). Conditional oculomotor learning: population vectors in the supplementary eye field. J. Neurophysiol. 78, 1166–1169. 9307145 10.1152/jn.1997.78.2.1166

[B26] Cooke D. Taylor C. Moore T. Graziano M. (2003). Complex movements evoked by microstimulation of the ventral intraparietal area. Proc. Natl. Acad. Sci. U.S.A. 100, 6163–6168. 10.1073/pnas.1031751100 12719522 PMC156343

[B27] Corneil B. Olivier E. Munoz D. (2002). Neck muscle responses to stimulation of monkey Superior Colliculus. I. Topography and manipulation of stimulation parameters. J. Neurophysiol. 88, 1980–1999. 10.1152/jn.00959.2001 12364523

[B28] Elsley J. K. Nagy B. Cushing S. L. Corneil B. D. (2007). Widespread presaccadic recruitment of neck muscles by stimulation of the primate frontal eye fields. J. Neurophysiol. 98, 1333–1354. 10.1152/jn.00386.2007 17625064

[B29] Freedman E. Sparks D. (1997). Eye-head coordination during head-unrestrained gaze shifts in rhesus monkeys. J. Neurophysiol. 77, 2328–2348. 9163361 10.1152/jn.1997.77.5.2328

[B30] Frey S. Mackey S. Petrides M. (2014). Cortico-cortical connections of areas 44 and 45B in the macaque monkey. Brain Lang. 131, 36–55. 10.1016/j.bandl.2013.05.005 24182840

[B31] Funahashi S. (2014). Saccade-related activity in the prefrontal cortex: its role in eye movement control and cognitive functions. Front. Integr. Neurosci. 8:54. 10.3389/fnint.2014.00054 25071482 PMC4074701

[B32] Fuster J. (1997). The Prefrontal Cortex: Anatomy, Physiology, and Neuropsychology of the Frontal Lobes. New York, NY: Raven.

[B33] Fuster J. (2008). The Prefrontal Cortex. London: Elsevier.

[B34] Fuster J. Bodner M. Kroger J. (2000). Cross-modal and cross-temporal association in neurons of frontal cortex. Nature 405, 347–351. 10.1038/35012613 10830963

[B35] Gandhi N. Sparks D. (2001). Experimental control of eye and head positions prior to head-unrestrained gaze shifts in monkey. Vision Res. 41, 3243–3254. 10.1016/S0042-6989(01)00054-2 11718770 PMC3655329

[B36] Gerbella M. Belmalih A. Borra E. Rozzi S. Luppino G. (2010). Cortical connections of the macaque caudal ventrolateral prefrontal areas 45A and 45B. Cereb. Cortex 20, 141–168. 10.1093/cercor/bhp087 19406905

[B37] Gottlieb J. (2013). Slicing a pie is no piece of cake. Nat. Neurosci. 16, 1364–1366. 10.1038/nn.3520 24067288

[B38] Graziano M. Taylor C. Moore T. (2002a). Complex movements evoked by microstimulation of precentral cortex. Neuron 34, 841–851. 10.1016/S0896-6273(02)00698-0 12062029

[B39] Graziano M. Taylor C. Moore T. Cooke D. (2002b). The cortical control of movement revisited. Neuron 36, 349–362. 10.1016/S0896-6273(02)01003-6 12408840

[B40] Groh J. M. Trause A. S. Underhill A. M. Clark K. R. Inati S. (2001). Eye position influences auditory responses in primate inferior colliculus. Neuron 29, 509–518. 10.1016/S0896-6273(01)00222-7 11239439

[B41] Gu C. Corneil B. D. (2014). Transcranial magnetic stimulation of the prefrontal cortex in awake nonhuman primates evokes a polysynaptic neck muscle response that reflects oculomotor activity at the time of stimulation. J. Neurosci. 34, 14803–14815. 10.1523/JNEUROSCI.2907-14.2014 25355232 PMC6608421

[B42] Hage S. R. Nieder A. (2013). Single neurons in monkey prefrontal cortex encode volitional initiation of vocalizations. Nat. Commun. 4:2409. 10.1038/ncomms3409 24008252

[B43] Judge S. Richmond B. Chu F. (1980). Implantation of magnetic search coils for measurement of eye position: an improved method. Vision Res. 20, 535–538. 10.1016/0042-6989(80)90128-5 6776685

[B44] Kampe K. K. Frith C. D. Frith U. (2003). “Hey John”: signals conveying communicative intention toward the self activate brain regions associated with “mentalizing,” regardless of modality. J. Neurosci. 23, 5258–5263. 12832550 10.1523/JNEUROSCI.23-12-05258.2003 PMC6741156

[B45] Kelly C. Uddin L. Q. Shehzad Z. Margulies D. S. Castellanos F. X. Milham M. P.. (2010). Broca's region: linking human brain functional connectivity data and non-human primate tracing anatomy studies. Eur. J. Neurosci. 32, 383–398. 10.1111/j.1460-9568.2010.07279.x 20662902 PMC3111969

[B46] Knight T. A. (2012). Contribution of the frontal eye field to gaze shifts in the head-unrestrained rhesus monkey: neuronal activity. Neuroscience 225, 213–236. 10.1016/j.neuroscience.2012.08.050 22944386 PMC3482142

[B47] Knight T. A. Fuchs A. F. (2007). Contribution of the frontal eye field to gaze shifts in the head-unrestrained monkey: effects of microstimulation. J. Neurophysiol. 97, 618–634. 10.1152/jn.00256.2006 17065243

[B48] Koechlin E. Ody C. Kouneiher F. (2003). The architecture of cognitive control in the human prefrontal cortex. Science 302, 1181–1185. 10.1126/science.1088545 14615530

[B49] Lanzilotto M. Perciavalle V. Lucchetti C. (2013a). Auditory and visual systems organization in Brodmann Area 8 for gaze-shift control: where we do not see, we can hear. Front. Behav. Neurosci. 7:198. 10.3389/fnbeh.2013.00198 24339805 PMC3857530

[B50] Lanzilotto M. Perciavalle V. Lucchetti C. (2013b). A new field in monkey's frontal cortex: Premotor Ear-Eye Field (PEEF). Neurosci. Biobehav. Rev. 37, 1434–1444. 10.1016/j.neubiorev.2013.05.010 23727051

[B51] Lanzilotto M. Perciavalle V. Lucchetti C. (2013c). Orienting movements in area 9 identified by long-train ICMS. Brain Struct. Funct. [Epub ahead of print]. 10.1007/s00429-013-0682-8 24337260

[B52] Lucchetti C. Lanzilotto M. Bon L. (2008). Auditory-motor and cognitive aspects in area 8B of macaque monkey's frontal cortex: a premotor ear-eye field (PEEF). Exp. Brain Res. 186, 131–141. 10.1007/s00221-007-1216-5 18038127

[B53] Luppino G. Rozzi S. Calzavara R. Matelli M. (2003). Prefrontal and agranular cingulate projections to the dorsal premotor areas F2 and F7 in the macaque monkey. Eur. J. Neurosci. 17, 559–578. 10.1046/j.1460-9568.2003.02476.x 12581174

[B54] Matelli M. Luppino G. Rizzolatti G. (1991). Architecture of superior and mesial area 6 and the adjacent cingulate cortex in the macaque monkey. J. Comp. Neurol. 311, 445–462. 10.1002/cne.903110402 1757597

[B55] Matsuzaka Y. Akiyama T. Tanji J. Mushiake H. (2012). Neuronal activity in the primate dorsomedial prefrontal cortex contributes to strategic selection of response tactics. Proc. Natl. Acad. Sci. U.S.A. 109, 4633–4638. 10.1073/pnas.1119971109 22371582 PMC3311351

[B56] Miller B. T. D'esposito M. (2005). Searching for “the Top” in top-down control. Neuron 48, 535–538. 10.1016/j.neuron.2005.11.002 16301170

[B57] Mitz A. Godschalk M. (1989). Eye-movement representation in the frontal lobe of rhesus monkeys. Neurosci. Lett. 106, 157–162. 10.1016/0304-3940(89)90219-X 2586821

[B58] Monteon J. Constantin A. Wang H. Martinez-Trujillo J. Crawford J. (2010). Electrical stimulation of the frontal eye fields in the head-free macaque evokes kinematically normal 3D gaze shifts. J. Neurophysiol. 104, 3462–3475. 10.1152/jn.01032.2009 20881198

[B59] Monteon J. Wang H. Martinez-Trujillo J. Crawford J. (2013). Frames of reference for eye-head gaze shifts evoked during frontal eye field stimulation. Eur. J. Neurosci. 37, 1754–1765. 10.1111/ejn.12175 23489744

[B60] Moschovakis A. Gregoriou G. Ugolini G. Doldan M. Graf W. Guldin W.. (2004). Oculomotor areas of the primate frontal lobes: a transneuronal transfer of rabies virus and [14C]-2-deoxyglucose functional imaging study. J. Neurosci. 24, 5726–5740. 10.1523/JNEUROSCI.1223-04.2004 15215295 PMC6729209

[B61] Mullette-Gillman O. D. A. Cohen Y. E. Groh J. M. (2005). Eye-centered, head-centered, and complex coding of visual and auditory targets in the intraparietal sulcus. J. Neurophysiol. 94, 2331–2352. 10.1152/jn.00021.2005 15843485

[B62] Newman J. Lindsley D. (1976). Single unit analysis of auditory processing in squirrel monkey frontal cortex. Exp. Brain Res. 25, 169–181. 10.1007/BF00234901 819284

[B63] O'reilly R. C. Noelle D. C. Braver T. S. Cohen J. D. (2002). Prefrontal cortex and dynamic categorization tasks: representational organization and neuromodulatory control. Cereb. Cortex 12, 246–257. 10.1093/cercor/12.3.246 11839599

[B64] Populin L. Tollin D. Yin T. (2004). Effect of eye position on saccades and neuronal responses to acoustic stimuli in the superior colliculus of the behaving cat. J. Neurophysiol. 92, 2151–2167. 10.1152/jn.00453.2004 15190094

[B65] Rauschecker J. Romanski L. (2011). Auditory cortical organization: evidence for functional streams, in The Auditory Cortex, eds Winer J. A. Schreiner C. E. (New York, NY: Springer), 99–116.

[B66] Remmel R. (1984). An inexpensive eye movement monitor using the scleral search coil technique. IEEE Trans. Biomed. Eng. 31, 388–390. 10.1109/TBME.1984.325352 6745975

[B67] Romanski L. Bates J. Goldman-Rakic P. (1999a). Auditory belt and parabelt projections to the prefrontal cortex in the rhesus monkey. J. Comp. Neurol. 403, 141–157. 9886040 10.1002/(sici)1096-9861(19990111)403:2<141::aid-cne1>3.0.co;2-v

[B68] Romanski L. Tian B. Fritz J. Mishkin M. Goldman-Rakic P. Rauschecker J. (1999b). Dual streams of auditory afferents target multiple domains in the primate prefrontal cortex. Nat. Neurosci. 2, 1131–1136. 10570492 10.1038/16056 PMC2778291

[B69] Schall J. Morel A. Kaas J. (1993). Topography of supplementary eye field afferents to frontal eye field in macaque: implications for mapping between saccade coordinate systems. Vis. Neurosci. 10, 385–393. 10.1017/S0952523800003771 7683486

[B70] Schall J. Morel A. King D. Bullier J. (1995). Topography of visual cortex connections with frontal eye field in macaque: convergence and segregation of processing streams. J. Neurosci. 15, 4464–4487. 7540675 10.1523/JNEUROSCI.15-06-04464.1995 PMC6577698

[B71] Schilbach L. Wohlschlaeger A. M. Kraemer N. C. Newen A. Shah N. J. Fink G. R.. (2006). Being with virtual others: neural correlates of social interaction. Neuropsychologia 44, 718–730. 10.1016/j.neuropsychologia.2005.07.017 16171833

[B72] Schlag J. Schlag-Rey M. (1987). Evidence for a supplementary eye field. J. Neurophysiol. 57, 179–200. 3559671 10.1152/jn.1987.57.1.179

[B73] Scudder C. Kaneko C. Fuchs A. (2002). The brainstem burst generator for saccadic eye movements. Exp. Brain Res. 142, 439–462. 10.1007/s00221-001-0912-9 11845241

[B74] Stanford T. Freedman E. Sparks D. (1996). Site and parameters of microstimulation: evidence for independent effects on the properties of saccades evoked from the primate superior colliculus. J. Neurophysiol. 76, 3360–3381. 8930279 10.1152/jn.1996.76.5.3360

[B75] Stein B. E. Clamann H. P. (1981). Control of pinna movements and sensorimotor register in cat superior colliculus. Brain Behav. Evol. 19, 180–192. 10.1159/000121641 7326575

[B76] Stein B. E. Stanford T. R. (2008). Multisensory integration: current issues from the perspective of the single neuron. Nat. Rev. Neurosci. 9, 255–266. 10.1038/nrn2331 18354398

[B77] Stepniewska I. Fang P. C. Kaas J. H. (2005). Microstimulation reveals specialized subregions for different complex movements in posterior parietal cortex of prosimian galagos. Proc. Natl. Acad. Sci. U.S.A. 102, 4878–4883. 10.1073/pnas.0501048102 15772167 PMC555725

[B78] Stepniewska I. Gharbawie O. A. Burish M. J. Kaas J. H. (2014). Effects of muscimol inactivations of functional domains in motor, premotor, and posterior parietal cortex on complex movements evoked by electrical stimulation. J. Neurophysiol. 111, 1100–1119. 10.1152/jn.00491.2013 24353298 PMC3949230

[B79] Stryker M. Schiller P. (1975). Eye and head movements evoked by electrical stimulation of monkey superior colliculus. Exp. Brain Res. 23, 103–112. 10.1007/BF00238733 1149845

[B80] Takahara D. Inoue K. Hirata Y. Miyachi S. Nambu A. Takada M.. (2012). Multisynaptic projections from the ventrolateral prefrontal cortex to the dorsal premotor cortex in macaques - anatomical substrate for conditional visuomotor behavior. Eur. J. Neurosci. 36, 3365–3375. 10.1111/j.1460-9568.2012.08251.x 22882424

[B81] Tehovnik E. Slocum W. Carvey C. (2003). Behavioural state affects saccadic eye movements evoked by microstimulation of striate cortex. Eur. J. Neurosci. 18, 969–979. 10.1046/j.1460-9568.2003.02798.x 12925023

[B82] Tehovnik E. Sommer M. (1997). Electrically evoked saccades from the dorsomedial frontal cortex and frontal eye fields: a parametric evaluation reveals differences between areas. Exp. Brain Res. 117, 369–378. 10.1007/s002210050231 9438704

[B83] Tehovnik E. Sommer M. Chou I. Slocum W. Schiller P. (2000). Eye fields in the frontal lobes of primates. Brain Res. Brain Res. Rev. 32, 413–448. 10.1016/S0165-0173(99)00092-2 10760550

[B84] Thier P. Andersen R. (1998). Electrical microstimulation distinguishes distinct saccade-related areas in the posterior parietal cortex. J. Neurophysiol. 80, 1713–1735. 9772234 10.1152/jn.1998.80.4.1713

[B85] Tu T. Keating E. (2000). Electrical stimulation of the frontal eye field in a monkey produces combined eye and head movements. J. Neurophysiol. 84, 1103–1106. 10938333 10.1152/jn.2000.84.2.1103

[B86] Urakawa S. Takamoto K. Ishikawa A. Ono T. Nishijo H. (2014). Selective medial prefrontal cortex responses during live mutual gaze interactions in human infants: an fNIRS study. Brain Topogr. 4, 4. [Epub ahead of print]. 10.1007/s10548-014-0414-2 25367848

[B87] Yang S. N. Heinen S. J. Missal M. (2008). The effects of microstimulation of the dorsomedial frontal cortex on saccade latency. J. Neurophysiol. 99, 1857–1870. 10.1152/jn.00119.2007 18216220

[B88] Yeterian E. Pandya D. (2010). Fiber pathways and cortical connections of preoccipital areas in rhesus monkeys. J. Comp. Neurol. 518, 3725–3751. 10.1002/cne.22420 20653031

[B89] Yeterian E. Pandya D. Tomaiuolo F. Petrides M. (2012). The cortical connectivity of the prefrontal cortex in the monkey brain. Cortex 48, 58–81. 10.1016/j.cortex.2011.03.004 21481342 PMC3161133

